# Detecting interaction networks in the human microbiome with conditional Granger causality

**DOI:** 10.1371/journal.pcbi.1007037

**Published:** 2019-05-20

**Authors:** Kumar Mainali, Sharon Bewick, Briana Vecchio-Pagan, David Karig, William F. Fagan

**Affiliations:** 1 Department of Biology, University of Maryland, College Park, Maryland, United States of America; 2 Research and Exploratory Development Department, Johns Hopkins University Applied Physics Laboratory, Laurel, Maryland, United States of America; DAL, CANADA

## Abstract

Human microbiome research is rife with studies attempting to deduce microbial correlation networks from sequencing data. Standard correlation and/or network analyses may be misleading when taken as an indication of taxon interactions because “correlation is neither necessary nor sufficient to establish causation”; environmental filtering can lead to correlation between non-interacting taxa. Unfortunately, microbial ecologists have generally used correlation as a proxy for causality although there is a general consensus about what constitutes a causal relationship: causes both precede and predict effects. We apply one of the first causal models for detecting interactions in human microbiome samples. Specifically, we analyze a long duration, high resolution time series of the human microbiome to decipher the networks of correlation and causation of human-associated microbial genera. We show that correlation is not a good proxy for biological interaction; we observed a weak negative relationship between correlation and causality. Strong interspecific interactions are disproportionately positive, whereas almost all strong intraspecific interactions are negative. Interestingly, intraspecific interactions also appear to act at a short timescale causing vast majority of the effects within 1–3 days. We report how different taxa are involved in causal relationships with others, and show that strong interspecific interactions are rarely conserved across two body sites whereas strong intraspecific interactions are much more conserved, ranging from 33% between the gut and right-hand to 70% between the two hands. Therefore, in the absence of guiding assumptions about ecological interactions, Granger causality and related techniques may be particularly helpful for understanding the driving factors governing microbiome composition and structure.

## Author summary

The human microbiome comprises thousands of microbial taxa, each of them potentially having interactions with many other taxa. Elucidating these interactions can be helpful for human health and beyond. The most conclusive approach for understanding microbial interactions would be myriads of controlled experiments in laboratory settings, each designed to test how taxa interact with one another. As this is a formidable challenge, an alternative strategy for constructing such an interaction network is a statistical framework employing formal models. Using conditional Granger causality, we demonstrate that correlation measures, which are very popular statistics in microbiome studies, are unreliable as proxies of microbial interactions. Overall, we found that strong interactions within a genus tended to be negative (e.g., competition), and they tended to occur on very short timescales (1–2 days). Many of such interactions were common across body sites. By contrast, strong interactions between genera tended to be positive (e.g., mutualism/facilitation) and were more evenly distributed over a range of timescales, up to the 20-day window that we considered. Very few of these interactions were conserved across body sites. We conclude that models of causality can be particularly useful in elucidating microbial interaction networks when laboratory investigations of interactions are impractical.

## Introduction

Human microbiome research is rife with studies attempting to deduce microbial correlation networks from sequencing data [1–5]. In many cases, the goal is to identify candidate species interactions, often with an aim to explore implications for human health. Pairs of taxa that exhibit negative correlations, for example, could act as probiotics, particularly if negative correlations are with pathogens. Likewise, pairs of taxa that exhibit positive correlations could provide an understanding of microbial succession—processes that can impact the composition, and thus ‘ecosystem services’ provided by the human microbiome [6]. Unfortunately, “correlation is neither necessary nor sufficient to establish causation” [7]. Thus, many of the microbial interactions identified from standard correlation and/or network analyses may be misleading, at least when taken as an indication of taxon interactions. A twin study of the human gut microbiome, for example, showed that most co-occurrence patterns are driven by host genetics, rather than by microbial taxon-taxon interactions [8].

More generally, co-occurrence/correlation in cross-sectional data can emerge from two fundamental processes that are mutually non-exclusive: species interactions and habitat or environmental filtering. Among species interactions, competition can lead to mutual exclusion [9] or negative correlations, whereas mutualism, commensalism or parasitism can establish positive correlations. Environmental filtering [10,11], by contrast, describes a process where existence is only possible for species with suitable traits. In this case, a pair of microbial species may co-occur because they have similar nutritional requirements and/or environmental tolerances. Conversely, species may be mutually absent if they differ in environmental requirements or tolerances. Thus, both positive and negative taxon-taxon correlations can emerge from distinct underlying processes. This makes it impossible to tease apart the drivers of correlation.

Even when species do interact, correlation analyses can still be problematic. First, non-linear dynamics can weaken correlations among interacting species. Indeed, simulation shows that even in a deterministic and dynamically coupled two species system, zero correlation is possible [7]. When this occurs, correlation metrics erroneously imply lack of causation. Consequently, inferring causation from correlation is risky, particularly in biological systems where non-linear dynamics are ubiquitous [7]. Second, when a true species interaction occurs with a time lag (e.g., a metabolite secreted by a species takes some time to diffuse through the environment, get absorbed by the recipient, go through synthetic pathways and elicit an effect in the recipient), correlation will not capture the interaction because it depends on instantaneous covariance between two time series.

Because of the problems with a correlation network approach, at least for the purposes of inferring taxon interactions, correlation networks should only be used when plausible interaction mechanisms can be identified for strongly correlated taxon pairs [2], and even then with the realization that many interactions may be missed because of nonlinearity and/or time-lagged responses. Unfortunately, because of the diversity of the human microbiome, and the fact that many taxa are only recently identified and thus poorly studied, the map of ecological, or even metabolic interactions among taxa is highly incomplete [12,13]. Instead, microbial ecologists have generally used correlation as a proxy for causality. In a recent study, for example, a number of correlation metrics and linear models were applied to a human microbiome dataset to decipher co-occurrence and co-exclusion networks [2]. Although the study only used cross-sectional data of presence-absence states, the researchers drew inferences about competition and exclusion between microbial clades. Another study [14] concluded that the highly centralized correlation networks in diseased versus healthy human oral microbiomes make diseased microbiomes more prone to a community shift in response to environmental change. This would be the case if central nodes control the structure of community stability. To make such an argument, however, it is necessary to assume that observed correlations stem from taxon interactions. Many additional studies have similarly assessed ecological interactions using taxon-taxon correlation/co-occurrence data [5,15–20], while others still have computed correlation/co-occurrence metrics but left their implications open for interpretation [4,21–25].

The difficulties associated with co-occurrence studies extend beyond theoretical interpretation. Laboratory studies on a simple but highly controlled closed system of three interacting microbial species [26] indicate that there are only two ways to robustly detect species interactions: biological replicates of a system at a given time and observation of a system over time. Because biological replicates of entire microbiomes are far from possible, this leaves analysis of time-series data as the sole method for accurately identifying pairs of interacting taxa.

Despite the challenges associated with identifying causality in sequencing data, there is nonetheless a general consensus about what constitutes a causal relationship: causes both precede and predict effects [27]. In keeping with the conclusions from the Hekstra and Leibler study [26], this implies a time element that is missing from most correlation analyses. The statistical approach known as Granger causality [7,28], however, establishes causation by predicting the current state of a system using past states. Specifically, variable X is the “Granger cause” of variable Y if and only if X uniquely improves predictability of Y—i.e., if forecasting the future states of Y based on its own past states is improved when the past states of X are also included in the model [28]. Cross prediction of time-series (prediction improvement of Y by adding X in the predictor set that already included Y plus prediction improvement of X by adding Y in the predictor set that already included X) is a powerful and intuitive approach to establish causality. Furthermore, unlike correlation and co-occurrence analyses, which are non-directional, Granger causality is directional. This is important in understanding species interactions because correlation, even when it captures true ecological interactions between X and Y, cannot determine whether X impacts Y or vice-versa, or whether the impact goes in both directions. This deficiency of correlation studies is corrected by Granger causality, which allows researchers to tease apart the cause and effect in a species interaction. Though Granger causality analysis has been applied to a variety of datasets, including those showing beta oscillations in cortical networks [29], societal crises in response to climate change [30] and the relationship between daily Dow Jones stock returns and percentage changes in New York Stock Exchange trading volume [31], it is almost non-existent in the analysis of human microbiomes [32] (although some researchers have pointed to it as a potentially powerful approach in that context [33,34]). In the only study that we came across that applied the models of Granger causality to microbiome data, Gibbons et al [32] analyzed human gut microbiome (four time series, one of them being the data we analyzed) using three lags in the model, with the main goal being to compare different methods of analysis and to identify the drivers of temporal dynamics of microbial abundance. We analyzed high resolution time series of the human microbiome at four body sites [35] using 20 lags in the causality models, to decipher strong positive and negative interactions, to identify those interactions that are short-acting versus long-acting, and to determine if interaction are conserved across body sites. Hence, compared to Gibbons et al. [32], our study not only addresses a different scientific question, but also goes beyond one body site to four and beyond short-timescales to both short- and long-timescales. In this study, we specifically seek to: (1) evaluate the relationship between correlation and causation, (2) decipher the networks of strong positive and negative interactions in all taxa-pairs at different time scales, and (3) determine if causal networks generalize across body sites.

## Results

One of the challenges of using Granger causality to explore ecological data is the interpretation of results. In particular, the output from a Granger causality analysis is a series of coefficients, each representing a different timescale, with different taxon pairs having different numbers of significant coefficients. To deal with this issue, we take several approaches. First, we consider general metrics, incorporating all coefficients. Then, to account for the fact that timescales differing by only a few days may represent the same or similar processes, we group time-lags into general, qualitative timescales, considering trends for ‘short’ (1–5 days) and ‘long’ (15–20 days) interactions, independent of precise day values.

### Correlation versus causality

**Fig 1:**
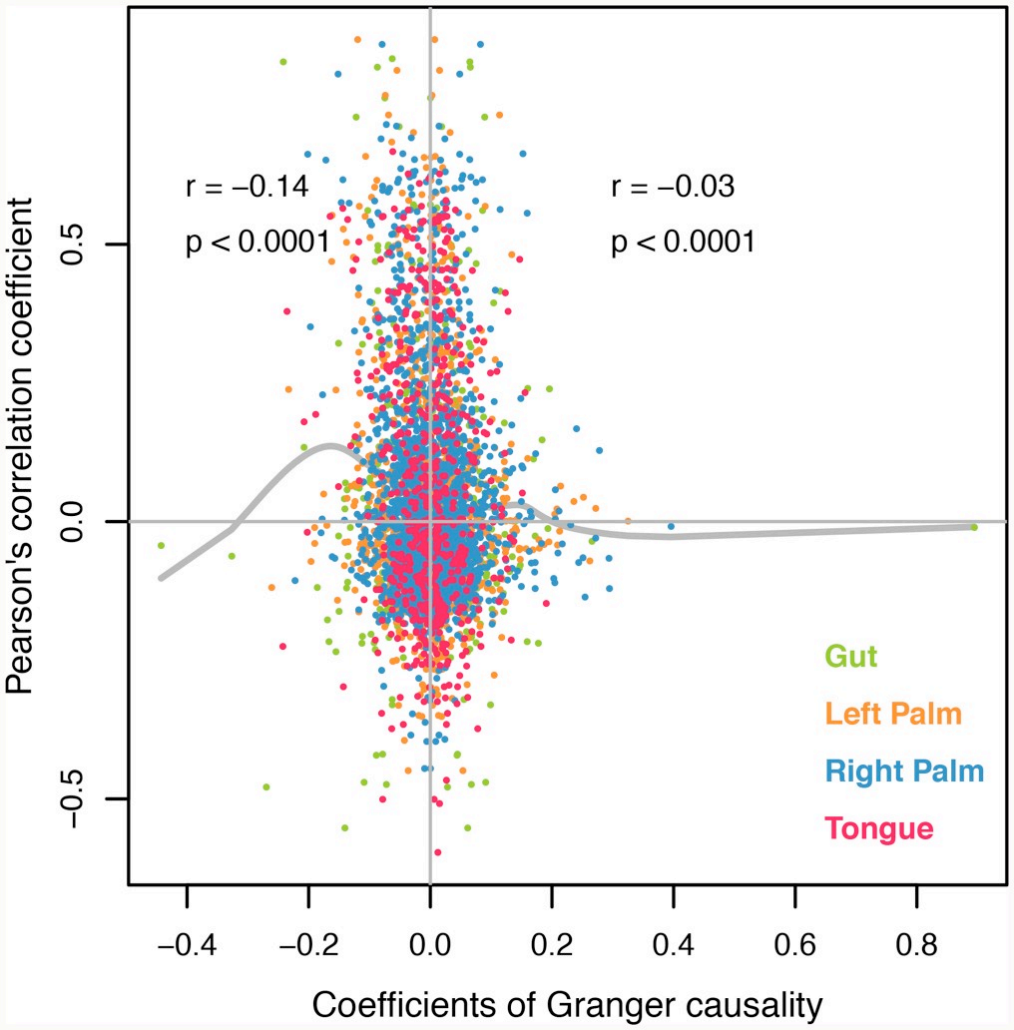
Granger causality vs Pearson’s correlation coefficient for all four body sites. Granger causality (GC) is expressed as change in abundance of response variable (in standard deviation) as the abundance of Granger-cause increases by 1 SD. Positive coefficients indicate positive effect of the Granger-cause on response in affecting abundance, and vice versa. For a given taxon-pair, the highest coefficient among all positive coefficients and the lowest one among all negative coefficients are shown in the figure. The relationship between Pearson’s correlation and Granger causality is shown separately for positive GC and negative GC along with separate splines.

When all taxon-pairs with significant Pearson’s correlation and Granger causality across all body sites were considered, we observed a weak negative relationship between Granger’s causality and Pearson’s correlation (Fig 1). If correlation inferred causality, then we would expect a strong positive relationship between Pearson’s correlation and Granger causality. Such a weak negative relationship indicates that correlation is either a completely unreliable metric of causation or it actually suggests a taxon-interaction in the opposite direction as compared to that indicated by Granger causality.

We further examine causality-correlation relationship within long- and short-timescales of each body site. For this analysis, we only consider interspecific interactions. The only taxon pairs that are excluded are those that exhibit conflicting Granger causality signs of interaction within the short or long timescale. Tables in S1 Table through S8 Table show the numbers of interspecific taxon interactions that are positive, negative or insignificant for Granger causality and simultaneously positive, negative or insignificant for Pearson correlations for short and long timescale interactions. Applying chi-square tests of independence to these data shows that Pearson and Granger models are not independent for short timescales. In particular, having a negative Pearson coefficient makes a taxa-pair far less likely to have a negative Granger coefficient. This is consistent with the aggregated analysis of all body sites for the correlation between Pearson’s correlation and Granger causality in Fig 1. Interestingly, although this same trend appears marginally significant at long timescales in the gut, it does not apply to long timescale interactions at other body sites, where there appears no discernable relationship between Pearson and Granger causality results (See S1–S8 Tables).

### Full quantitative analysis

When all the significant coefficients of Granger causality in each body site were examined collectively, vast majority of them are very small in magnitude (S1 Fig). Realizing that effect size can be important for ecological interpretation, we concentrate our results only to the top 5 percentile coefficients (“**strong coefficients**”, hereafter) for each of the positive and negative interactions in a body site. These strongest signals of causality were separately detected in each body site.

**Fig 2:**
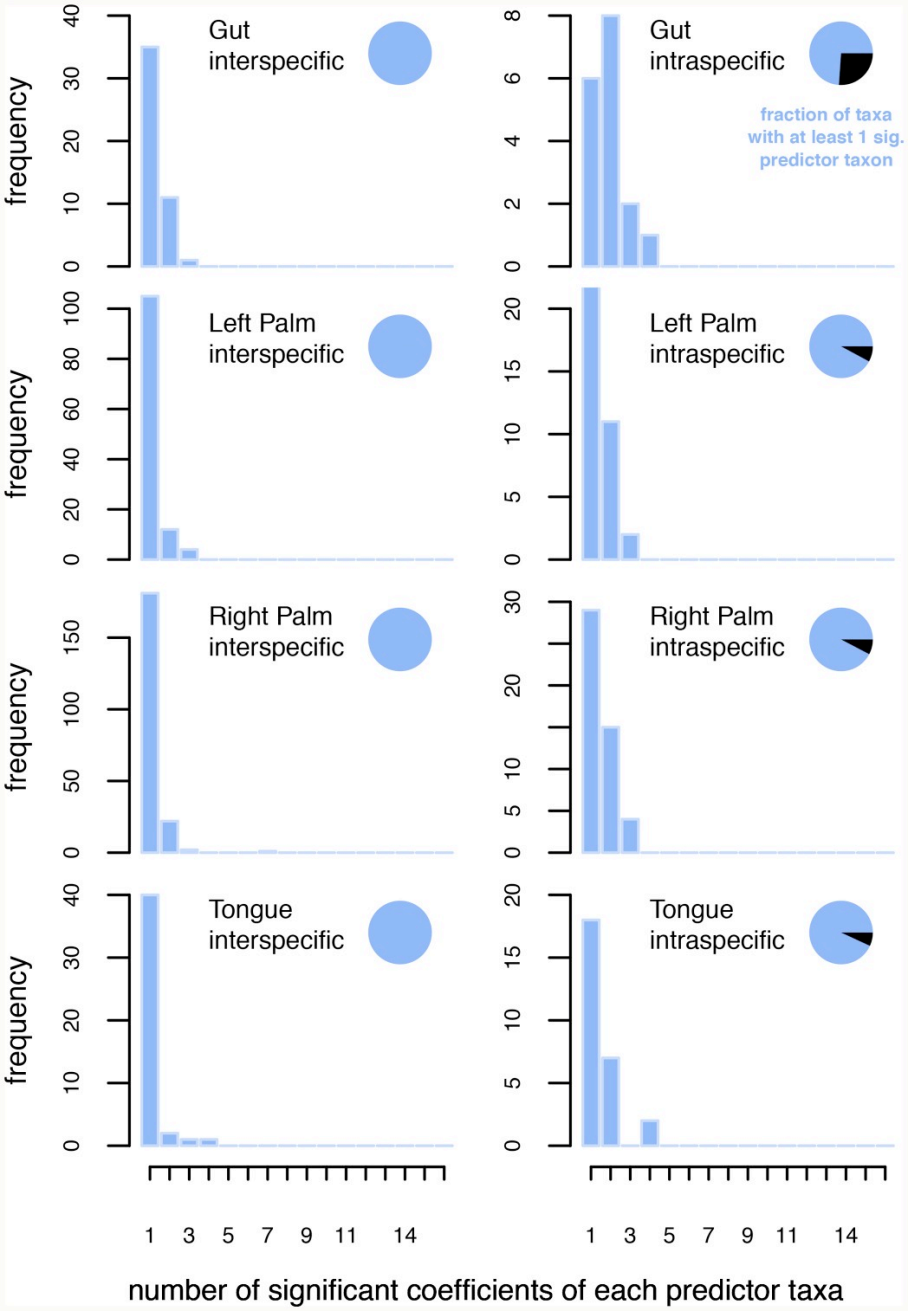
Number of strong coefficients of Granger causality for interspecific (left column) and intraspecific (right column) taxon pairs in the gut, and on the left-hand, right-hand and tongue. For a given response variable (taxon), the total number of significant coefficients of each predictor taxon was noted. Plots were created by the totality of such coefficients for all response variables. Inset pie-charts show the fraction of taxa with (colored) and without (black) at least one significant predictor.

Fig 2 shows the number of strong coefficients for all pairs of taxa at each body site for interspecific (left column) and intraspecific (right column) interactions (for this analysis we take taxon A → taxon B and taxon B → taxon A as separate pairs). Of all the taxa in a body site, at least three-fourths had at least one intraspecific significant predictor (inset pie chart). Every taxon had at least one interspecific significant predictor (inset pie chart). When a taxon is predicted by another taxon, in vast majority of the cases, only one coefficient/lag (not necessarily the first lag) was significant for interspecific interactions whereas up to two coefficients made the vast majority of intraspecific interactions. In both cases, a few taxa-pairs are involved with up to four significant coefficients although the highest number observed was seven. The fact that intraspecific taxon pairs have more strongest coefficients than interspecific interactions suggests that a larger number of timescales are important for intraspecific relative to interspecific interactions.

**Fig 3:**
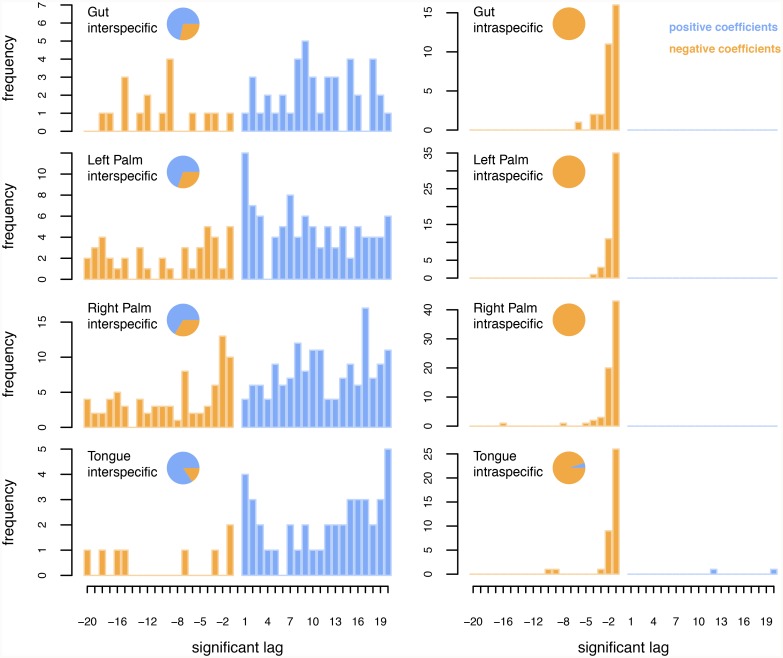
Frequency with which strong coefficients appear at different time-lags for interspecific interactions (left column) and for intraspecific interactions (right column). Coefficients are shown for the gut, left-hand, right-hand and tongue. For each panel, the negative axis reflects time-lags with negative coefficients, while the positive axis reflects time-lags with positive coefficients. Pie-charts adjacent to each panel show the fraction of coefficients that are positive (blue) versus negative (orange).

Fig 3 shows the frequency with which strongest coefficients for different time-lags appear in the models for each taxon pair at each body site. Interspecific interactions appear disproportionately positive and relatively evenly distributed across time-lags (Fig 3, left column, see adjacent pie-charts). By contrast, almost all of intraspecific interactions are negative (Fig 3, right column), and the vast majority of interactions occur on a 1-day timescale, with a small fraction extending to 2 days and very few at 3 days or beyond. This suggests that there is short-term, intraspecific suppression for a wide range of different taxa at all body sites.

**Fig 4:**
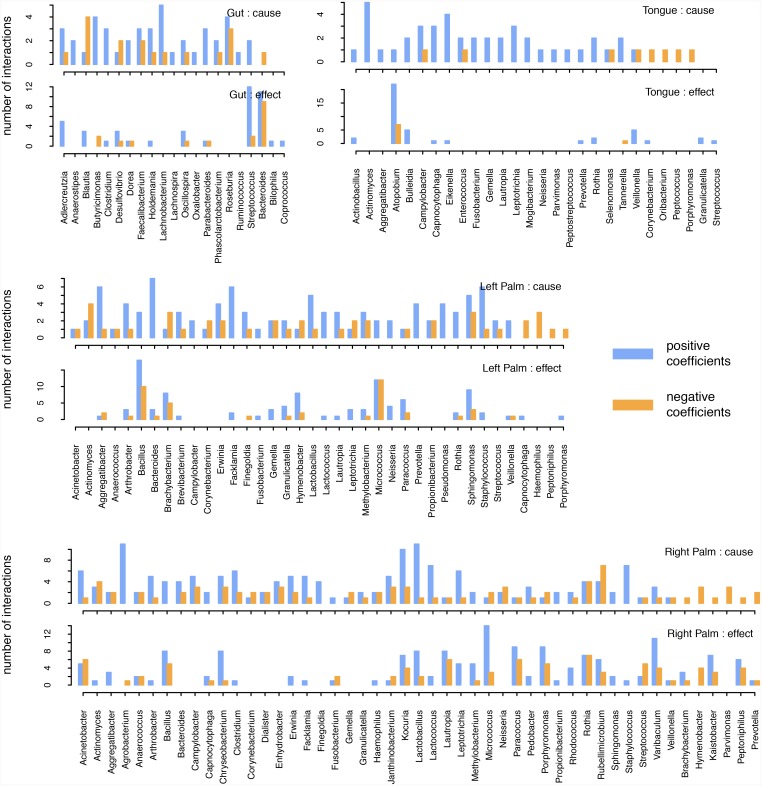
Total number of strong interspecific cause and effect interactions that are positive (blue) and negative (orange) in the gut and on the tongue, left-hand and right-hand. Results are shown for each genus with at least one significant interaction.

**Fig 5:**
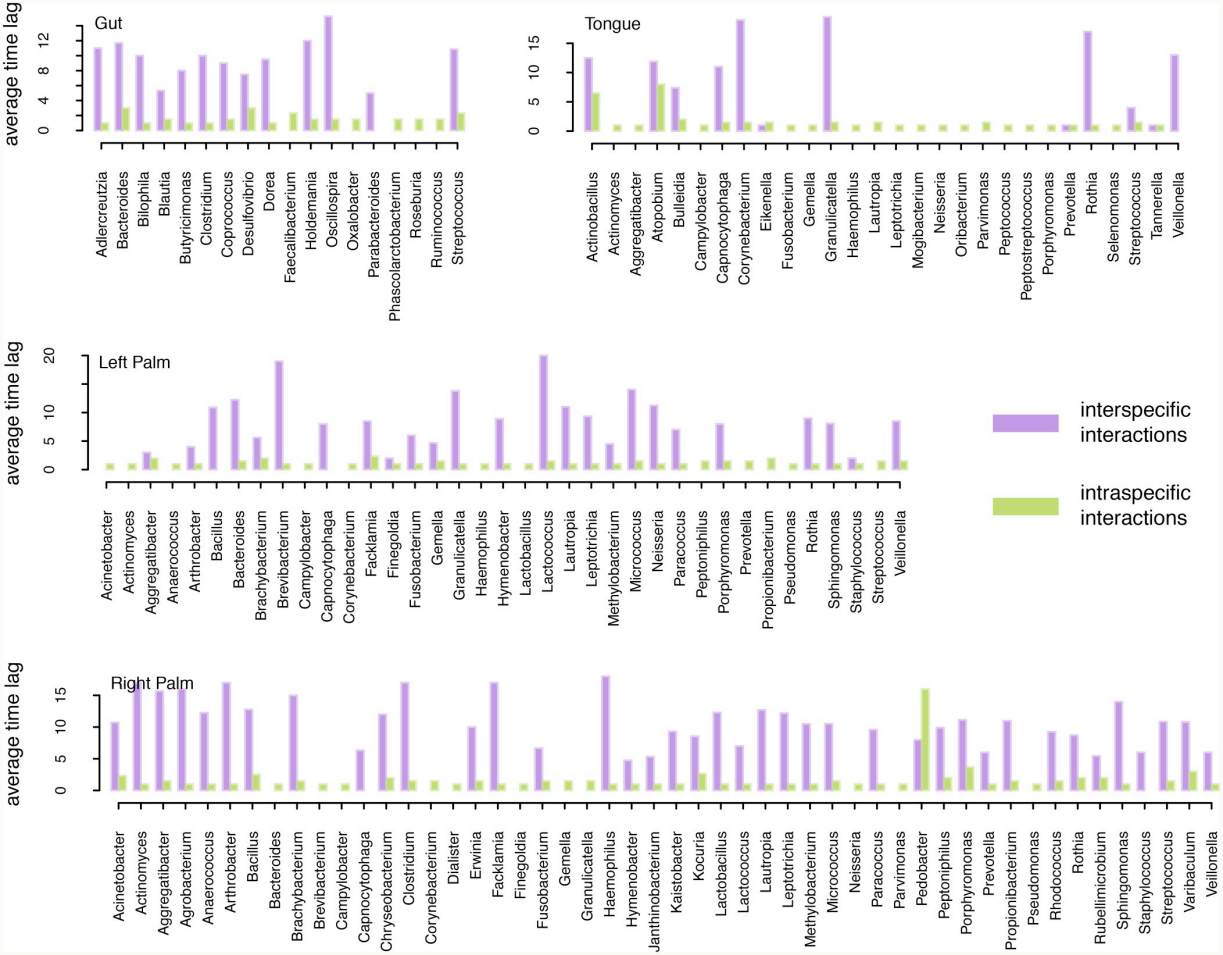
Average time-lag associated with all strong interspecific interactions (purple) and with all strong intraspecific interactions (green) in the gut and on the tongue, left-hand and right-hand. Results are shown for each genus with at least one significant interaction.

In Fig 4, we show how different taxa are involved in Granger causality relationships. Specifically, we show the number of positive/negative cause/effect interspecific interactions by genus. Meanwhile, Fig 5 shows the average time-lags associated with each taxon’s interspecific and intraspecific interactions respectively. As in Fig 3, Fig 5 reiterates the fact that interspecific interactions, even those that are relatively strong, occur over a range of timescales, whereas intraspecific interactions act primarily over short timescales. One notable exception to this trend is *Pedobacter* in right palm.

### Body-site comparisons across timescales

In S2 Fig (see S1 Text), we show a Venn diagram illustrating the number of shared taxa amongst different sets of body sites. The left- and right-hand are the most diverse, and also share the largest fraction of genera (73%). The gut is the least diverse and shares very few genera with the other three body sites (15%, 10% and 5% with the right-hand, left-hand and tongue respectively). This distribution of taxa constrains the number of interactions that can be conserved across body sites. However, even given these constraints, conservation is remarkably low. Amongst strong interspecific interactions, for example, there is only one that is conserved across two body sites, and that is a positive interaction between *Micrococcus* and *Veillonella* on the left- and right-hand at a timescale of 20 days. (See S1 Text for an analysis of conservation for all interactions, both weak and strong). Strong intraspecific interactions are much more conserved, ranging from 33% between the gut and right-hand to 70% between the two hands.

**Fig 6:**
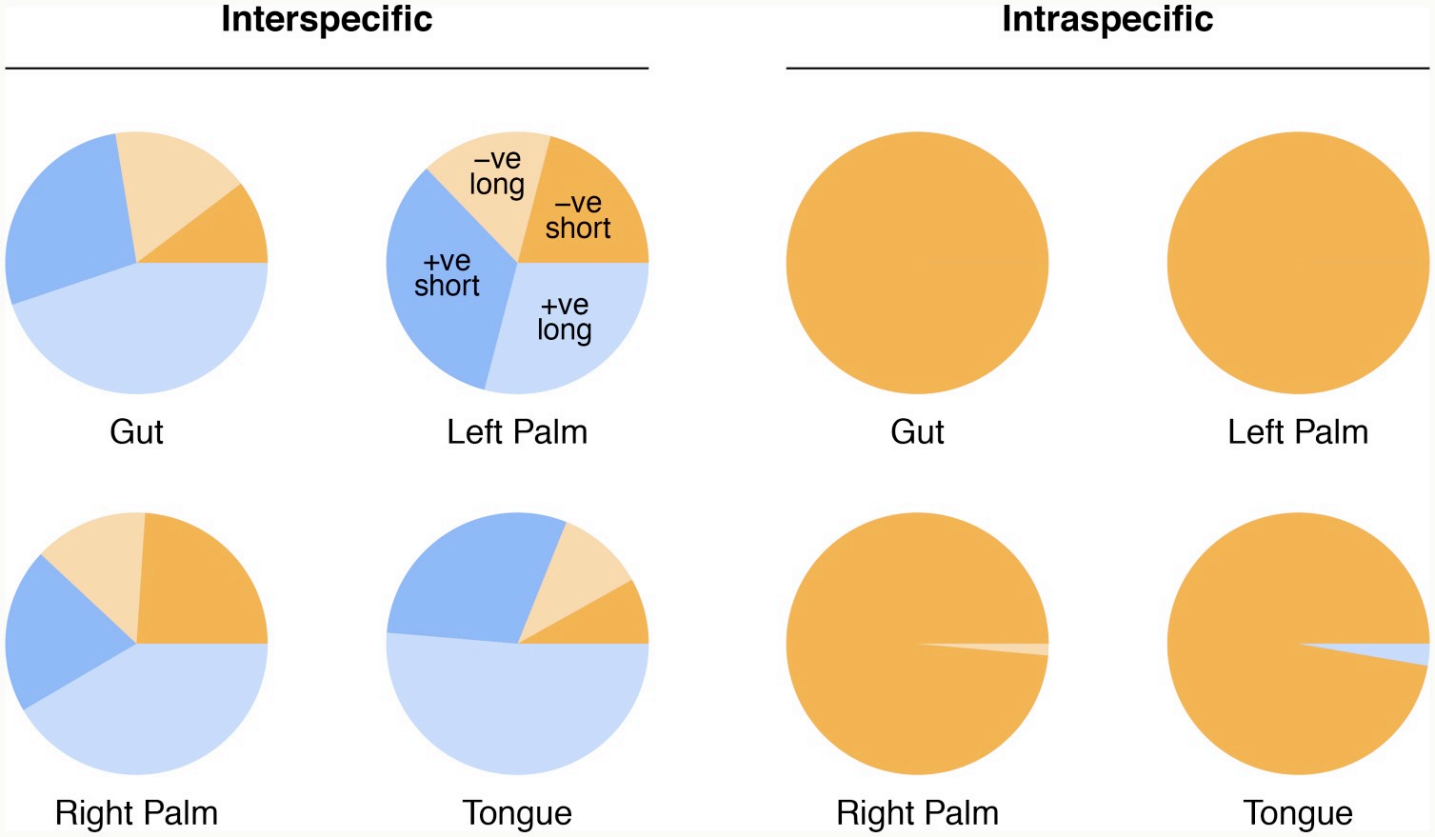
Fraction of short/long and negative/positive interactions with strong coefficients. The fractions are shown separately for interspecific and intraspecific interactions within each body site.

One of the problems with considering individual time-lags separately is that it makes observing conserved interactions across sites very difficult. This is because conservation must be precise. Countering such precision is the fact that sampling almost certainly did not occur at the same time each day (for the gut, for instance, sampling is restricted to the timing of bowel movements). Likewise, similar processes may occur at somewhat different timescales at different body sites, particularly if resource availability or other environmental factors cause organisms to grow at different rates. For this reason, we now turn our attention to a qualitative analysis, where we consider any time-lag between 1–5 days as a ‘short’ interaction, and any time-lag between 15–20 days as a ‘long’ interaction. We ignore ‘short’ and/or ‘long’ interactions for any taxon pair with multiple coefficients at the short or long timescale with opposite signs. Fig 6 shows how interactions break down for strong interactions with coefficients in the top 5% by magnitude (see S2 Text for an analysis of all coefficients). As in Fig 3, we see that strong interspecific interactions are predominantly positive, and this is particularly true for long timescales. By contrast, strong intraspecific interactions are almost uniformly short and negative, again consistent with Fig 3. Although, no strong interspecific coefficients are conserved across three body sites, a few are conserved across the two hands. These are shown in Table 1. All strong intraspecific coefficients are conserved.

**Table 1:** Strong qualitative interactions conserved across the left-hand and right-hand. Taxa pairs that are conserved both for interaction and timescale are shown.

Cause	Effect	Timescale	Interaction
*Aggregatibacter*	*Bacillus*	long	negative
*Arthrobacter*	*Micrococcus*	short	positive
*Erwinia*	*Paracoccus*	short	positive
*Haemophilus*	*Veillonella*	short	negative
*Lactobacillus*	*Micrococcus*	long	positive
*Lautropia*	*Paracoccus*	long	positive
*Veillonella*	*Micrococcus*	long	positive

## Discussion

Strong positive or negative correlations between taxa can emerge as a result of species interactions [9] or via environmental filtering [10,11]. With existing research, which primarily focuses on basic assessment of correlation patterns [1–5,36,37], it is difficult to determine the underlying causes of observed species distributions. Highlighting the challenges associated with teasing out species interactions from standard microbiome datasets, Berry and Wider [38] simulated multi-species microbial communities by generating interaction patterns with generalized Lotka-Volterra dynamics. They found that co-occurrence networks can be a proxy for interaction networks under certain conditions; however, with significant habitat filtering, the interpretation of co-occurrence becomes problematic. Unfortunately, few microbiome correlation studies explicitly discuss the pitfalls associated with using correlation/co-occurrence metrics to infer ecological interactions [12,39]. A prior human microbiome study explicitly showed that correlation is not a reliable metric of interaction [39]. The conclusion of that study, however, comes from a simulation experiment with a set of specified conditions and not from the analysis of real-world data. Here, by analyzing four of the longest and densest time series from the human microbiome, we show that correlation is not a reliable proxy of ecological interaction (measured with Granger causality) in human microbiome, and indeed the two measures are weakly negatively related across the dataset (Fig 1). As Granger causality is beginning to see its application in microbiome studies [32], many types of interactions can be elucidated with these causal models. Unfortunately, ground-truthing of the causal models is highly limited at present because of very few in-vitro studies (e.g., [40]).

Sometimes, knowledge of the biological system under investigation can help to inform models of community interactions; however, this is not always the case. Particularly for complex microbiomes, a deeper understanding of biology is often insufficient to resolve conflicting hypotheses. A genome-scale metabolic modeling of the human gut microbiome, for example, found that species that strongly compete with each other (i.e., species with highly similar nutritional profiles) tend to co-occur, whereas species pairs that co-occur least often have dissimilar nutritional profiles, suggesting that environmental filtering is the main driver of community structure [12]. In contrast, a subsequent community metabolic model assembled from models of species level metabolic exchanges analyzed >800 microbial communities and found that species interactions, in particular metabolic dependencies, are a “major driver of species co-occurrence” [41].

One solution for trying to identify species interactions in complex communities is to move beyond correlation (*t =* 0) to focus on causation (i.e., *t* > 0 correlation). Stated simply, this is the temporal dependence of one taxon on another. Although many studies identify a ‘core human microbiota’ which is stable over long timescales [35,42–44], there is still significant short timescale variation [35,42], making it possible to examine causal relationships within community dynamics. In the present study, we used the longest available time series of human microbiome dynamics [35] to elucidate causal networks among constituents of the human microbiome.

Interestingly, we find that strong interspecific interactions tend to be positive (see Fig 3). This is in opposition to the few experimental studies that exist. For example, Foster and Bell [45] analyzed overall respiration, and found that the great majority of interactions are net negative. Similarly, by culturing artificial microbial communities of 1–12 species for 60 generations and comparing community yield against the sum of species’ yields in monoculture, Fiegna *et al*. [46] demonstrated that interactions are, in general, negative, although they also showed that interactions become less negative over time.

Interestingly, our analysis also shows a tendency towards more positive interactions over longer timescales (see Fig 6), at least for strong interactions. One reason we may detect more positive interactions overall is that our analysis is based on data from communities in vivo, where cell-cell adhesion and formation of complex biofilms may be important for persistence. Physical requirements for persistence in environments like the gut or oral cavity may outweigh metabolic competition in terms of the net degree of mutualism/facilitation amongst community members.

Our finding that intraspecific interactions tend to occur on short timescales and tend to be strongly negative (see Fig 3) suggests that individual populations are kept in check not by other bacterial taxa, but rather by factors intrinsic to themselves. This might be resource limitation; however, for this to be the case, resource use would have to lead to overcompensatory dynamics, such that a large population on one day led to a crash the following day. Though not impossible, a more likely explanation is natural enemies, such as phages. In essence, then, the picture that emerges for maintenance of biodiversity in the human microbiome is one of a temporal Jansen-Connell effect. In particular, when any single population begins to dominate, ‘density-dependent’, host-specific pathogens attack the population, leading to collapse. An analogous way to view our findings is from a Lotka-Volterra competition framework. Specifically, we find stable coexistence among large numbers of microbes because each member of a pair of species inhibits its own population growth more strongly than it inhibits the population growth of others [47].

Comparing body sites, we find very few shared interactions, even when accounting for taxonomic differences in community composition among the gut, hands and tongue. Indeed, for our full quantitative analysis with all time-lags (see S1 Text, S9 Table), we find <1% of interspecific coefficients conserved across the tongue, and two hands, which share a total of 18 genera. Intraspecific models are more conserved, although even for these, only 13–20% of coefficients are shared across three or more sites (see S1 Text, S9 Table). When we use a qualitative analysis, results improve, although even here, very few interactions remain across multiple body sites (see Table 1 and S2 Text). Examining qualitative interactions that are conserved across the tongue, left- and right-hands, it is interesting to note that there are clusters of negative interactions that align with known spatial segregation of specific taxa, suggesting the potential for niche competition. For example, there is inhibition between *Leptotrichia*, *Capnocytophaga* and *Fusobacterium* (see S2 Text, S11 Table), all of which are predominantly found in a wide band just inside the periphery of ‘hedge-hog’ structures in plaque [48]. Meanwhile, we see another distinct cluster of negative interactions involving *Rothia*, *Haemophilus*, *Neisseria* and *Veillonella* (see S2 Text, S11 Table). Notably, *Rothia*, *Veillonella* and *Haemophilus* are members of ‘cauliflower’ structures within plaque [48]. Meanwhile, *Rothia* and *Neisseria* are both early colonizers of oral cavity surfaces, again suggesting niche overlap where competition might occur [49].

Contrasting Granger causality and more standard correlation analysis with Pearson Correlation coefficients, it is interesting to note that there does appear to be some relationship between the two for short timescale interactions. Contrary to what is expected, this relationship is, however, negative. That is, positive Pearson correlation coefficients are more likely to have negative Granger causality coefficients, at least at short timescales. Long timescale Granger causality results do not appear to be related to Pearson correlation except, perhaps, minimally in the gut. The explanation for these observations is unclear, but may point to a tendency to compete with other taxa that share a similar environmental niche.

Two previous studies [33,50] have taken a time series regression approach to the same microbiome dataset that we analyzed. However, neither qualify as a Granger causality analysis, differing from ours in two key ways. First, their methods did not integrate cross-prediction (where a given taxon is predicted by both its own lagged states and the suspected Granger cause, and vice versa). In our model, a causal relationship is established only when the cause significantly improves prediction of a model that already includes lagged states of the response variable. Second, their analysis included only the first lag. There is no reason to believe interactions completely disappear after one day, and indeed we found significant terms out to 20 days.

### Conclusion

Biological interactions of microbial communities are so little known that mapping out the architecture of interactions is currently a formidable challenge [19,51]. Therefore, statistical analysis of correlation/co-occurrence networks, which are increasingly available because of high-throughput cross-sectional sequencing, could serve as a first approximation of biological interactions. However, making the leap from co-occurrence data to causality requires statistical tools that can actually elucidate causation, which is not possible with a simple correlation approach. Indeed, correlation networks may have no relationship to interaction networks. Therefore, in the absence of guiding assumptions about ecological interactions, Granger causality and related techniques may be particularly helpful for understanding the driving factors governing microbiome composition and structure.

## Methods

### Data source and processing

Granger causality is, in essence, a method for determining taxon correlations through time. As a consequence, a minimum requirement is a well-resolved microbiome time series. For our work, we used data from Caporaso et al. [35], which is the longest human microbiome time series to date. In brief, this dataset comprises 16S sequences for all bacterial taxa over a period of 336–373 days from four body sites (Table 2). Although the original study considered two human subjects—one male and one female—we focused on the male data, since this subject was followed over a longer period of time. To simplify our analysis, we performed all calculations on genus level taxonomic assignments. Further, we only considered genera with a measurable abundance in at least 90% of the time series. However, even for these taxa, up to 10% of data points may be absences. Because absences can be problematic for Granger causality analyses, we replaced absences with randomly sampled small abundances (a random number generated between 10^−5^ and 10^−3^ of the mean of the time series). The assumption is that genera that are present on >90% of days were not actually missing on the other days, but were instead not detected because their abundances fell below the detection threshold [35]. For all of our analyses, we first calculated relative (i.e., normalized) abundances. However, because genera can differ in their relative abundances by several orders of magnitude, we performed our analyses on standard deviation changes in abundance. Specifically, we standardized the relative abundance of each genus by its own mean and standard deviation across the time series. Finally, we removed stochastic and deterministic trends by first-differencing. Analyses were performed separately for each of the four body sites.

**Table 2:** Characteristics of body sites and model. Each Genus in a body site was predicted by a sum of its own lags as well as that of all other Genus. A separate model was built to predict each Genus by itself (endogeneous variable) and all the other Genus (exogeneous variables). Each Genus in a body site was therefore provided with the same set of predictors but the final model retained different set of predictors (Genus and lags).

Body site	Length of time series	Total number of Genus in the body site	Number of Genus that were saved by Lasso in the final model	Number of parameters initially considered to predict each Genus	Number of parameters saved by Lasso in the final model of each predicted Genus	Fraction of all parameters (lags) that were retained by Lasso in the final model
Gut	336	23	2–22	460	2–108	0.30
Left palm	365	38	1–38	760	1–111	0.25
Right palm	359	52	1–43	1040	1–73	0.20
Tongue	373	29	1–29	580	1–111	0.23

### Granger causality analysis

The original Granger causality model included only two time series—the potential Granger cause and the response—as predictors [28]. Two types of processes can lead to spurious causation in such pair-wise analysis of causality. First, when taxon A interacts with taxa B and C at different time lags, but taxa B and C are causally independent, an analysis showing causal relationship between taxa B and C is incorrect. Second, when the flow of causation is from taxon A to B and then from taxon B to C, a detection of causation between taxa A and C is spurious. These two situations—the first one called as “differentially delayed driving” and the second one as “sequential driving”–have been tested in the traditional, pair-wise framework of Granger causation [52]. In a simulation experiment of 500 realizations with each of them being 100 timesteps long, Chen et al. [52] showed that the classical pair-wise Granger causality identifies spurious causation resulting from both aforementioned processes as the true causation. However, when Chen et al. performed a multivariate or conditional Granger causality analysis (including all three time series in the model), the spurious causations detected by the pair-wise Granger analysis were removed whereas all true causations were retained [52]. Realizing the power of multivariate methods, several multivariate extensions of Granger causality [53–56] have been developed recently, and we have implemented one of these multivariate approaches here.

The superiority of multivariate Granger causality over the traditional, pair-wise approach can be explained by statistical theory. In a model with two predictors, the coefficients reflect the effect of one predictor when the other predictor variable is also included in the model and is held constant [57]. This partial effect of a predictor is the unique effect of the variable in predicting the response variable [58]. As a consequence, the spurious causations detected using a pair-wise Granger causality are correctly eliminated by using a multivariate/conditional Granger causality analysis. Since both the statistical insights, as well as prior simulation experiments, show that multivariate or conditional Granger analysis eliminates the cases of spurious causations that plague the traditional pair-wise analysis of Granger causation, we applied a multivariate approach of Granger causality in this current study.

Another advantage of multivariate analysis is a dramatic reduction in the number of hypothesis tests and false positives. In a standard Granger causality analysis, the number of hypotheses tested would be 2*(n2) for *n* taxa. In our dataset, for example, this would yield 253 hypotheses for the gut microbial community and 1326 for the right palm. With the conventional approach of hypothesis testing with alpha of 0.05, we can potentially have up to 66 false positive cases of causation in the right palm alone (i.e., significant causation identified when no causation exists). By using a multivariate approach, however, the number of hypotheses tested is reduced to 23 and 52 for gut and right palm, respectively, and the number of false positives in the right palm is reduced to 3.

To take advantage of the multivariate approach, we implemented multivariate/conditional analysis of Granger causality in this study. To construct multivariate models, we assumed that the relative abundance of any particular genus could be dependent on all other genera in the community; we then considered 20 time-lags (for a maximum lag of approximately 20 days). We chose this time range because we found that lags beyond 15–17 days rarely improve the models. Because we wanted to include multiple time lags as predictors, we were faced with a large over-parameterization problem. For example, each response genus in the gut had 460 predictors, while each response genus on the right palm had 1040 predictors (Table 2). To reduce parameters and ensure predictive power, we applied what is known as a least absolute shrinkage and selection operator (LASSO) [59]. LASSO performs both regularization and variable selection by shrinking large coefficients and eliminating smaller ones. This results in a simpler model with better interpretability and stability. To perform LASSO, we tested a range of shrinkage parameters, selecting the best shrinkage parameter based on it, and yielding a model with minimum prediction error when tested against independent data (cross-validation).

**Fig 7:**
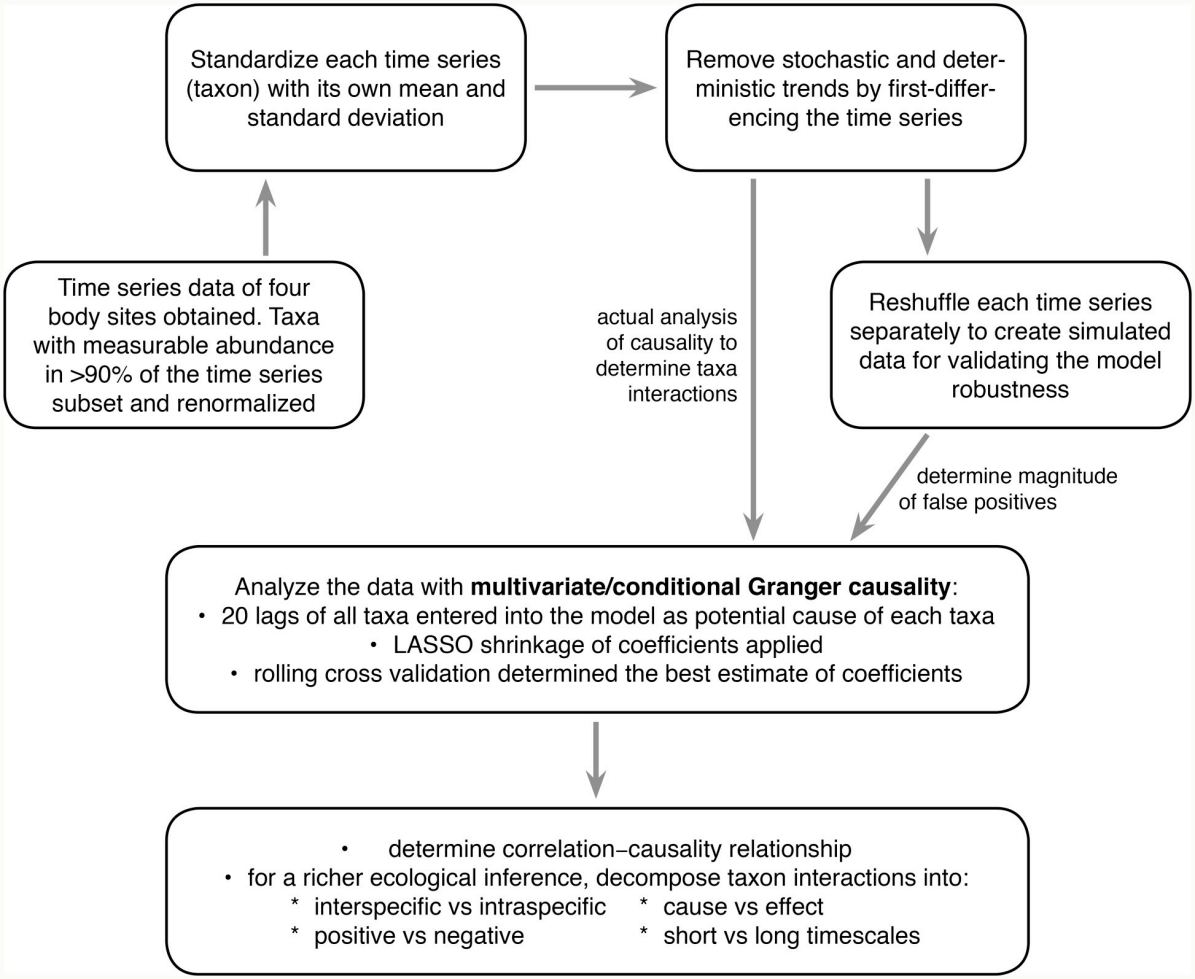
Flow chart of the methods and models we employed. In one of the first applications of causal models in microbiome interaction network analysis, we used conditional/multivariate model of Granger causality which eliminates spurious causations but retains the true ones (this is in contrast to the traditional more famous pair-wise model of Granger causality which detects spurious causations). LASSO shrinkage and rolling cross validation of the model makes it simpler and more robust. This overall method is novel in microbiome network analysis. The novelty in the findings of our study is that we show conclusively as the first report that correlation does not inform causality at all in human microbiome. For a richer ecological inference, we decomposed the taxa interactions into (1) interspecific vs intraspecific, (2) positive vs negative, (3) cause vs effect, and (4) short vs long timescales.

Completion of model building was achieved in three steps that included rolling cross-validation [60,61]. Specifically, we built a model using only the first one-third of the time series. A range of penalty parameters were then tested by sequentially adding one observation at a time from the second third of the time series; the best penalty parameter was selected so as to minimize the mean-squared forecast error. Finally, independent validation of the model was performed using the final third of the time series. This method of rolling cross-validation assisted LASSO shrinkage of the model eliminated about three-quarters of the total parameters (Table 2). Whereas cross-validation is considered a gold standard of model evaluation, there is an additional reason to apply such methods to microbiome datasets: taxon associations in human gut microbiota have been shown to vary in strength over time [34]. This makes cross-validation of the model crucial for model evaluation and selection. Granger causality analysis was performed in R (version 3.4.1) using the “BigVAR” package [62]. For a visual presentation of the sequence of the overall methods in the study, we developed a flow chart (Fig 7).

Although prior simulation [52] and statistical insight [57,58] (discussed above) validate the multivariate/conditional Granger causality for its strengths of identifying true causation and eliminating spurious causation detected by pair-wise Granger analysis, we went a step further and determined the robustness of our model by reshuffling the data we analyzed and determining how many causations are detected in the randomized data. When the time series are randomized, the temporal relationship between lags should disappear and so, ideally speaking, no causations should be detected (some false positives are always allowed because of the non-zero Type I error [alpha] of the model). We calculated the number of strong coefficients detected from reshuffled time series as well as from real data. We applied the same threshold to determine strong coefficients in the actual and randomized data for a given body site (see “Full quantitative analysis” under the Results section for definition). The number of taxa-pairs with strong coefficients detected using the reshuffled data was a mere 7% of the number of strong coefficients detected in the actual data (S12 Table). This gives us high confidence that, despite the complexities in the model and data, the composite modeling approach we used, which goes beyond traditional Granger analysis, has detected true signal, as expected under a robust statistical model with reasonable tolerance of false positives.

Although we detected 41 interacting taxa-pairs with strong coefficients using the reshuffled data across all four body sites, only five of those taxa- pairs were found in the results of the actual data. We eliminated those five taxa-pairs from the results presented in this study.

### The problem of compositional structure in data

Relative abundance data suffers from the problem of compositionality which can yield spurious correlation. There have been attempts to deal with this problem. Faust et al [2] proposed a method to generate an appropriate null distribution of correlation by permutation and renormalization, accounting for the compositional structure of the data. Although this approach yields a null appropriate for compositional data in a pairwise analysis, it does not alter the estimate itself, which makes it irrelevant to our multivariate analysis. Another study by Friedman and Alm [15] went a step further and proposed a new estimate of correlation. However, their mathematical formulation of this metric was developed for pairwise comparison and there is no straightforward way to include this in a multivariate regression framework. Although our analysis cannot rely on these recently developed approaches to minimize the impact of compositionality, we have employed a stringent LASSO shrinkage of the model that eliminated three-fourths of the coefficients, retaining only the very strongest and most highly significant relationships. On top of that, most of the results we have shown are for the strongest 5% of all the significant results. Additionally and importantly, our analysis does not suffer from one of the key problems of compositional data: singularity of the design matrix for the regression yielding no unique solution to the ordinary least square problem [39,63]. In our analysis, because on average only one fourth of the parameters are used, the regression model is well-defined.

After applying all the procedures, we tested the results against that of reshuffled time series of the actual data. This test shows that the false positives of our results is likely to be about 7% on average across body sites. Whereas we do not have a straightforward way of determining the extent compositionality affected our result, the reshuffled results give us high confidence in our results.

## Supporting information

S1 Text Number of shared taxa amongst different sets of body sites and conserved taxon interactions across body sites. (DOCX)

S2 Text Interaction categories for long vs short, positive vs negative, interspecific vs intraspecific relationships within each body site and pairwise comparison of body sites. (DOCX)

S1 Table Correlation vs short timescale causality in the gut. Number of taxon pairs with positive, negative and insignificant interactions for Pearson correlation and short timescale Granger causality models of the gut.
(DOCX)

S2 Table Correlation vs long timescale causality in the gut. Number of taxon pairs with positive, negative and insignificant interactions for Pearson correlation and long timescale Granger causality models of the gut.
(DOCX)

S3 Table Correlation vs short timescale causality on the left-hand. Number of taxon pairs with positive, negative and insignificant interactions for Pearson correlation and short timescale Granger causality models of the left-hand.
(DOCX)

S4 Table Correlation vs long timescale causality on the left-hand. Number of taxon pairs with positive, negative and insignificant interactions for Pearson correlation and long timescale Granger causality models of the left-hand.
(DOCX)

S5 Table Correlation vs short timescale causality on the right-hand. Number of taxon pairs with positive, negative and insignificant interactions for Pearson correlation and short timescale Granger causality models of the right-hand.
(DOCX)

S6 Table Correlation vs long timescale causality on the right-hand. Number of taxon pairs with positive, negative and insignificant interactions for Pearson correlation and long timescale Granger causality models of the right-hand.
(DOCX)

S7 Table Correlation vs short timescale causality on the tongue. Number of taxon pairs with positive, negative and insignificant interactions for Pearson correlation and short timescale Granger causality models of the tongue.
(DOCX)

S8 Table Correlation vs long timescale causality on the tongue. Number of taxon pairs with positive, negative and insignificant interactions for Pearson correlation and long timescale Granger causality models of the tongue.
(DOCX)

S9 Table Comparison of Granger causality models across body sites. (DOCX)

S10 Table Conserved interactions including both interspecific (black) and intraspecific (red) interactions. (DOCX)

S11 Table Qualitative interactions conserved across the tongue, left- and right-hand. (DOCX)

S12 Table Validating model with randomized data. Number of strong coefficients detected from randomized data as well as from real data.
(DOCX)

S1 Fig Coefficients of Granger causality for each body site. Effects outside of the orange bars correspond to the strongest 5% of effects in positive and negative interactions.
(TIF)

S2 Fig Shared taxa amongst the body sites, and causally related taxon-pairs that are shared and unique across the body sites. (A) Venn diagram showing the number of shared genera amongst the gut, left-hand, right-hand and tongue; (B,C) Pie charts for pairwise combinations of body sites illustrating the number of time-lags with significant, taxon-specific coefficients that are unique to one or other body site (solid) or else shared between body sites (striped). For each chart, we include only those taxa found on both body sites being compared (see A) and treat positive and negative coefficients separately (i.e., to be classified as a shared time-lag, the sign must be the same). Individual panels are as follows: (B) interspecific interactions considering all time-lags from 1 to 20 days, (C) intraspecific interactions considering all time-lags from 1 to 20 days.
(TIF)

S3 Fig Interaction categories for long vs short, positive vs negative, interspecific vs intraspecific relationships. (A,B) Fraction of taxon pairs at the four body sites that exhibit no interaction (white), consistent long and/or short interactions (solid), only interactions between 11–14 days (wavy lines) and only short and/or long interactions with conflicting signs for (A) interspecific interactions and (B) intraspecific interactions. (C,D) Fraction of consistent short and/or long interacting taxon pairs with various combinations of negative and positive short and long interactions for (C) interspecific interactions and (D) intraspecific interactions.
(TIF)

S4 Fig Pie charts for pairwise combinations of body sites illustrating the number of time-lags with significant, taxon-specific coefficients that are unique to one or other body site (solid) or else shared between body sites (striped). For each chart, we include only those taxa found on both body sites being compared (see S2 Fig, panel A) and treat positive and negative coefficients separately (i.e., to be classified as a shared time-lag, the sign must be the same). Individual panels are as follows: (A) interspecific interactions considering only ‘short’ and ‘long’ timescales and (B) intraspecific interactions considering only ‘short’ and ‘long’ timescales.
(TIF)

## References

[1] Mainali KP, Bewick S, Thielen P, Mehoke T, Breitwieser FP, Paudel S, et al Statistical analysis of co-occurrence patterns in microbial presence-absence datasets. PLoS One. 2017;12 10.1371/journal.pone.0187132 29145425 PMC5689832

[2] Faust K, Sathirapongsasuti JF, Izard J, Segata N, Gevers D, Raes J, et al Microbial co-occurrence relationships in the human microbiome. PLoS Comput Biol. 2012;8: e1002606–e1002606. 10.1371/journal.pcbi.1002606 22807668 PMC3395616

[3] Arumugam M, Raes J, Pelletier E, Nature DLP-, 2011 U. Enterotypes of the human gut microbiome. Nature. 2011;473: 174–180. 10.1038/nature09944 21508958 PMC3728647

[4] Zhang Z, Geng J, Tang X, Fan H, Xu J, Wen X, et al Spatial heterogeneity and co-occurrence patterns of human mucosal-associated intestinal microbiota. ISME J. 2014;8: 881–893. 10.1038/ismej.2013.185 24132077 PMC3960530

[5] Oh J, Freeman AF, NISC Comparative Sequencing Program NCS, Park M, Sokolic R, Candotti F, et al The altered landscape of the human skin microbiome in patients with primary immunodeficiencies. Genome Res. 2013;23: 2103–14. 10.1101/gr.159467.113 24170601 PMC3847779

[6] Turnbaugh PJ, Hamady M, Yatsunenko T, Cantarel BL, Duncan A, Ley RE, et al A core gut microbiome in obese and lean twins. Nature. 2009;457: 480 10.1038/nature07540 19043404 PMC2677729

[7] Sugihara G, May R, Ye H, Hsieh C -h., Deyle E, Fogarty M, et al Detecting Causality in Complex Ecosystems. Science (80-). 2012;338: 496–500. 10.1126/science.1227079 22997134

[8] Goodrich JK, Waters JL, Poole AC, Sutter JL, Koren O, Blekhman R, et al Human genetics shape the gut microbiome. Cell. 2014;159: 789–799. 10.1016/j.cell.2014.09.053 25417156 PMC4255478

[9] Cody ML, Diamond JM. Ecology and evolution of communities. Belknap Press of Harvard University Press; 1975.

[10] Bazzaz FA. Habitat Selection in Plants. Am Nat. 1991;137: S116–S130. 10.1086/285142

[11] Woodward FI, Diament AD. Functional Approaches to Predicting the Ecological Effects of Global Change. Funct Ecol. 1991;5: 202 10.2307/2389258

[12] Levy R, Borenstein E. Metabolic modeling of species interaction in the human microbiome elucidates community-level assembly rules. Proc Natl Acad Sci. 2013;110: 12804–12809. 10.1073/pnas.1300926110 23858463 PMC3732988

[13] Klitgord N, Segre D. Environments that induce synthetic microbial ecosystems. PLoS Comput Biol. 2010;6: e1001002 10.1371/journal.pcbi.1001002 21124952 PMC2987903

[14] Duran-Pinedo AE, Paster B, Teles R, Frias-Lopez J. Correlation Network Analysis Applied to Complex Biofilm Communities. Gilbert JA, editor. PLoS One. 2011;6: e28438 10.1371/journal.pone.0028438 22163302 PMC3233593

[15] Friedman J, Alm EJ. Inferring Correlation Networks from Genomic Survey Data. von Mering C, editor. PLoS Comput Biol. 2012;8: e1002687 10.1371/journal.pcbi.1002687 23028285 PMC3447976

[16] Kittelmann S, Seedorf H, Walters WA, Clemente JC, Knight R, Gordon JI, et al Simultaneous amplicon sequencing to explore co-occurrence patterns of bacterial, archaeal and eukaryotic microorganisms in rumen microbial communities. PLoS One. 2013;8: e47879 10.1371/journal.pone.0047879 23408926 PMC3568148

[17] King AJ, Farrer EC, Suding KN, Schmidt SK. Co-occurrence patterns of plants and soil bacteria in the high-alpine subnival zone track environmental harshness. Front Microbiol. 2012;3: 347 10.3389/fmicb.2012.00347 23087675 PMC3469205

[18] Williams RJ, Howe A, Hofmockel KS. Demonstrating microbial co-occurrence pattern analyses within and between ecosystems. Front Microbiol. 2014;5: 358 10.3389/fmicb.2014.00358 25101065 PMC4102878

[19] Widder S, Besemer K, Singer GA, Ceola S, Bertuzzo E, Quince C, et al Fluvial network organization imprints on microbial co-occurrence networks. Proc Natl Acad Sci. 2014;111: 12799–12804. 10.1073/pnas.1411723111 25136087 PMC4156742

[20] Lee MS, Oh S, Tang H. Characterization of microbial associations in human oral microbiome. Biomed Mater Eng. 2014;24: 3737–3744. 10.3233/BME-141202 25227089

[21] Findley K, Oh J, Yang J, Conlan S, Deming C, Meyer JA, et al Topographic diversity of fungal and bacterial communities in human skin. Nature. 2013;498: 367–370. 10.1038/nature12171 23698366 PMC3711185

[22] Oh J, Byrd AL, Deming C, Conlan S, Kong HH, Segre JA, et al Biogeography and individuality shape function in the human skin metagenome. Nature. 2014;514: 59–64. 10.1038/nature13786 25279917 PMC4185404

[23] Oh J, Conlan S, Polley EC, Segre JA, Kong HH. Shifts in human skin and nares microbiota of healthy children and adults. Genome Med. 2012;4: 77 10.1186/gm378 23050952 PMC3580446

[24] Tu Q, Zhou X, He Z, Xue K, Wu L, Reich P, et al The diversity and co-occurrence patterns of N 2-fixing communities in a CO 2-enriched grassland ecosystem. Microb Ecol. 2016;71: 604–615. 10.1007/s00248-015-0659-7 26280746

[25] Kamneva OK. Genome composition and phylogeny of microbes predict their co-occurrence in the environment. PLoS Comput Biol. 2017;13: e1005366 10.1371/journal.pcbi.1005366 28152007 PMC5313232

[26] Hekstra DR, Leibler S. Contingency and statistical laws in replicate microbial closed ecosystems. Cell. 2012;149: 1164–1173. 10.1016/j.cell.2012.03.040 22632978

[27] Wiedermann W, von Eye A. Statistics and causality: Methods for applied empirical research. John Wiley & Sons; 2016.

[28] Granger CWJ. Investigating Causal Relations by Econometric Models and Cross-spectral Methods. Econometrica. 1969;37: 424 10.2307/1912791

[29] Brovelli A, Ding M, Ledberg A, Chen Y, Nakamura R, Bressler SL. Beta oscillations in a large-scale sensorimotor cortical network: directional influences revealed by Granger causality. Proc Natl Acad Sci. 2004;101: 9849–9854. 10.1073/pnas.0308538101 15210971 PMC470781

[30] Zhang DD, Lee HF, Wang C, Li B, Pei Q, Zhang J, et al The causality analysis of climate change and large-scale human crisis. Proc Natl Acad Sci. 2011;108: 17296–17301. 10.1073/pnas.1104268108 21969578 PMC3198350

[31] Hiemstra C, Jones JD. Testing for linear and nonlinear Granger causality in the stock price-volume relation. J Finance. 1994;49: 1639–1664.

[32] Gibbons SM, Kearney SM, Smillie CS, Alm EJ. Two dynamic regimes in the human gut microbiome. PLoS Comput Biol. Public Library of Science; 2017;13: e1005364. 10.1371/journal.pcbi.1005364 PMC5340412 28222117

[33] Trosvik P, de Muinck EJ, Stenseth NC. Biotic interactions and temporal dynamics of the human gastrointestinal microbiota. ISME J. 2015;9: 533–541. 10.1038/ismej.2014.147 25148482 PMC4331571

[34] Faust K, Lahti L, Gonze D, de Vos WM, Raes J. Metagenomics meets time series analysis: Unraveling microbial community dynamics. Curr Opin Microbiol. 2015;25: 56–66. 10.1016/j.mib.2015.04.004 26005845

[35] Caporaso JG, Lauber CL, Costello EK, Berg-Lyons D, Gonzalez A, Stombaugh J, et al Moving pictures of the human microbiome. Genome Biol. 2011;12: R50. 10.1186/gb-2011-12-5-r50 21624126 PMC3271711

[36] Qin J, Li R, Raes J, Arumugam M, Burgdorf KS, Manichanh C, et al A human gut microbial gene catalogue established by metagenomic sequencing. Nature. 2010;464: 59–65. 10.1038/nature08821 20203603 PMC3779803

[37] Huttenhower C, Gevers D, Knight R, Abubucker S, Badger JH, Chinwalla AT, et al Structure, function and diversity of the healthy human microbiome. Nature. 2012;486: 207–214. 10.1038/nature11234 22699609 PMC3564958

[38] Berry D, Widder S. Deciphering microbial interactions and detecting keystone species with co-occurrence networks. Front Microbiol. 2014;5: 10–3389. 24904535 10.3389/fmicb.2014.00219 PMC4033041

[39] Fisher CK, Mehta P. Identifying keystone species in the human gut microbiome from metagenomic timeseries using sparse linear regression. PLoS One. 2014;9: e102451 10.1371/journal.pone.0102451 25054627 PMC4108331

[40] Das P, Ji B, Kovatcheva-Datchary P, Bäckhed F, Nielsen J. In vitro co-cultures of human gut bacterial species as predicted from co-occurrence network analysis. PLoS One. Public Library of Science; 2018;13: e0195161. 10.1371/journal.pone.0195161 PMC5877883 29601608

[41] Zelezniak A, Andrejev S, Ponomarova O, Mende DR, Bork P, Patil KR. Metabolic dependencies drive species co-occurrence in diverse microbial communities. Proc Natl Acad Sci. 2015;112: 6449–6454. 10.1073/pnas.1421834112 25941371 PMC4443341

[42] Oh J, Byrd AL, Park M, Kong HH, Segre JA. Temporal Stability of the Human Skin Microbiome. Cell. 2016;165: 854–866. 10.1016/j.cell.2016.04.008 27153496 PMC4860256

[43] Faith JJ, Guruge JL, Charbonneau M, Subramanian S, Seedorf H, Goodman AL, et al The long-term stability of the human gut microbiota. Science (80-). 2013;341: 1237439 10.1126/science.1237439 23828941 PMC3791589

[44] Schloissnig S, Arumugam M, Sunagawa S, Mitreva M, Tap J, Zhu A, et al Genomic variation landscape of the human gut microbiome. Nature. 2013;493: 45–50. 10.1038/nature11711 23222524 PMC3536929

[45] Foster KR, Bell T. Competition, not cooperation, dominates interactions among culturable microbial species. Curr Biol. 2012;22: 1845–1850. 10.1016/j.cub.2012.08.005 22959348

[46] Fiegna F, Moreno-Letelier A, Bell T, Barraclough TG. Evolution of species interactions determines microbial community productivity in new environments. ISME J. 2015;9: 1235–1245. 10.1038/ismej.2014.215 25387206 PMC4409166

[47] Silvertown J. Plant coexistence and the niche. Trends Ecol Evol. 2004;19: 605–611.

[48] Welch JLM, Rossetti BJ, Rieken CW, Dewhirst FE, Borisy GG. Biogeography of a human oral microbiome at the micron scale. Proc Natl Acad Sci. 2016;113: E791—E800. 10.1073/pnas.1522149113 26811460 PMC4760785

[49] Jenkinson HF. Beyond the oral microbiome. Environ Microbiol. 2011;13: 3077–3087. 10.1111/j.1462-2920.2011.02573.x 21906224

[50] Trosvik P, de Muinck EJ. Ecology of bacteria in the human gastrointestinal tract—identification of keystone and foundation taxa. Microbiome. BioMed Central; 2015;3: 44 10.1186/s40168-015-0107-4 PMC4601151 26455879

[51] Kurtz ZD, Müller CL, Miraldi ER, Littman DR, Blaser MJ, Bonneau RA. Sparse and Compositionally Robust Inference of Microbial Ecological Networks. von Mering C, editor. PLOS Comput Biol. 2015;11: e1004226 10.1371/journal.pcbi.1004226 25950956 PMC4423992

[52] Chen Y, Bressler SL, Ding M. Frequency decomposition of conditional Granger causality and application to multivariate neural field potential data. J Neurosci Methods. Elsevier; 2006;150: 228–237. 10.1016/j.jneumeth.2005.06.011 16099512

[53] Siggiridou E, Kugiumtzis D. Granger causality in multivariate time series using a time-ordered restricted vector autoregressive model. IEEE Trans Signal Process. 2016;64: 1759–1773.

[54] Basu S, Shojaie A, Michailidis G. Network Granger causality with inherent grouping structure. J Mach Learn Res. 2015;16: 417–453. PMC8278320 34267606

[55] Lozano AC, Abe N, Liu Y, Rosset S. Grouped graphical Granger modeling for gene expression regulatory networks discovery. Bioinformatics. 2009;25: i110—i118. 10.1093/bioinformatics/btp199 19477976 PMC2687953

[56] Shojaie A, Michailidis G. Discovering graphical Granger causality using the truncating lasso penalty. Bioinformatics. 2010;26: i517—i523. 10.1093/bioinformatics/btq377 20823316 PMC2935442

[57] Kutner MH, Nachtsheim CJ, Neter J, Li W, others. Applied linear statistical models. McGraw-Hill New York; 2005.

[58] Weisberg S. Applied linear regression. John Wiley & Sons; 2005.

[59] Tibshirani R. Regression shrinkage and selection via the lasso. J R Stat Soc Ser B. JSTOR; 1996; 267–288.

[60] Bańbura M, Giannone D, Reichlin L. Large Bayesian vector auto regressions. J Appl Econom. 2010;25: 71–92.

[61] Nicholson WB, Matteson DS, Bien J. VARX-L: Structured regularization for large vector autoregressions with exogenous variables. Int J Forecast. 2017;33: 627–651.

[62] Nicholson W, Matteson D, Bien J. BigVAR: Tools for Modeling Sparse High-Dimensional Multivariate Time Series. arXiv Prepr arXiv170207094. 2017;

[63] Jackson DA. Compositional data in community ecology: the paradigm or peril of proportions? Ecology. Wiley Online Library; 1997;78: 929–940.

