# Supplementary material for: Detecting interaction networks in the human microbiome with conditional Granger causality
Source: PLoS Comput Biol. 2019 May 20;15(5):e1007037. doi: 10.1371/journal.pcbi.1007037 (PMC6544333; doi:10.1371/journal.pcbi.1007037)
Supplement: S1 Text — (DOCX) [file pcbi.1007037.s001.docx]

**S1 Text. Number of shared taxa amongst different sets of body sites and conserved taxon interactions across body sites.**

In S9 Table, we outline the number of possible and actual (i.e., taxon pairs that have at least one significant coefficient of Granger causality at all sites) taxon interactions that are or could be shared across sites. Per interacting taxon pair, we also show the average number of significant time-lags (summed over all sites), the average number of significant time-lags that are conserved (including sign) across all sites, the average number of time-lags that are conserved across all sites except for a change of sign, the average number of time-lags that show a change of sign between at least two sites (for pairs of sites, this is identical to the average number of time-lags that are conserved except for a change of sign; for more than two sites, this includes time-lags that change sign between two sites, and are not present at the third or fourth site), and finally, the average number of time-lags that are not conserved across all sites (i.e., not present, regardless of sign, in at least one site). In S10 Table, we show all conserved interactions across sets of body sites.

From S9 Table, it is clear that, at least for interspecific interactions, the degree of model conservation across sites is very low. Indeed, the number of shared coefficients between sites is not much more than would be expected by chance based on the average number of coefficients per interaction at each site independently (see the last 4 rows of S9 Table for interspecific interactions). Further impressing this point is the fact that the fraction of conserved time-lags is similar to the fraction of time-lags that undergo sign reversal across all pairwise combinations of body sites. However, the degree of model conservation across sites for intraspecific interactions is much higher, and well above what might be expected by chance or what is seen for terms with sign reversal.

S2 Fig expand on the data in S9 Table, showing pie-charts for each pairwise body site combination. Specifically, we illustrate the number of significant time-lags that are unique to each body site (solid) or shared between body sites (striped), summed across all taxon pairs present at both body sites in the pair. As in S9 Table, for interspecific interactions, models across body sites are strikingly different, with the fraction of shared time-lags ranging from 0% between the gut and left-hand to 7.1% between the gut and right-hand. Models for intraspecific interactions are more conserved, with the fraction of shared time-lags ranging from 24% between the gut and right-hand to 30.4% between the gut and left-hand (notice that percentages differ slightly between the pie-charts and S9 Table because the pie-charts are based on total number coefficients, whereas S9 Table is based on average number of coefficients per interaction). In total, there are only three interspecific interactions that are conserved across three body sites. Interestingly, all of these involve effects on *Rothia*. Specifically, we find a positive effect on *Rothia* due to *Corynebacterium* (1 day) and *Veillonella* (17 days) and a negative effect on *Rothia* due to *Neisseria* (2 days). These interactions are present on the left-hand, right-hand and tongue, but not in the gut where none of these four species is present. Unfortunately, none of the coefficients for the interactions conserved across three body sites is large (i.e., top 5%).
