# Supplementary material for: Detecting interaction networks in the human microbiome with conditional Granger causality
Source: PLoS Comput Biol. 2019 May 20;15(5):e1007037. doi: 10.1371/journal.pcbi.1007037 (PMC6544333; doi:10.1371/journal.pcbi.1007037)
Supplement: S2 Text — (DOCX) [file pcbi.1007037.s002.docx]

**S2 Text. Interaction categories for long vs short, positive vs negative, interspecific vs intraspecific relationships within each body site and pairwise comparison of body sites.**

S3 Fig shows a breakdown of the number of taxon pairs that exhibited consistent long, consistent short or consistent long and short interactions (A,B), as well as the fraction of interacting taxa with different combinations of positive and/or negative interactions and short and long timescales (C,D). Importantly, even for our short and long timescale analysis we retain interactions amongst slightly less than 50% of interspecific taxon pairs and >78% of intraspecific taxon pairs. For interspecific taxon pairs, ~40% exhibit only short timescale interactions, with fairly even partitioning between negative and positive effects. Slightly fewer taxa, ~35% exhibit only long timescale interactions. Again, the partitioning between negative and positive effects is similar, although for long timescale interactions, all body sites show more positive interactions than negative interactions. Approximately 25% of taxon pairs exhibit both short and long timescale interactions, and again, this fraction is fairly evenly divided amongst the four combinations of positive and negative effects. For intraspecific interactions, the vast majority (≥50%) exhibit only short negative interactions. The remainders exhibit short negative interactions, along with long interactions with the long interactions being fairly evenly partitioned between negative and positive effects.

As expected, by combining all time-lags between 1-5 days and 15-20 days, we are able to find more interactions that are conserved across multiple body sites. This is shown in S4 Fig, where we again use pie charts to illustrate the number of interaction coefficients that are unique versus shared across pairs of body sites. Comparing S2 Fig and S4 Fig, for example, we see that there is a dramatic increase in the number of interspecific interaction coefficients shared across body sites. For intraspecific interactions the outcome is even more dramatic. In fact, in all body site combinations except for the gut/right-hand the vast majority of coefficients are shared across sites.

Once again, because there is so little taxon overlap between the gut and the other three body sites, interspecific interactions are shared across a maximum of three body sites: the tongue, the right-hand and the left-hand. Conserved three-site interactions are shown in S11 Table. Interestingly, all conserved short time-scale interactions are negative, whereas the two conserved long time-scale interactions are positive.
