# Supplementary material for: Detecting interaction networks in the human microbiome with conditional Granger causality
Source: PLoS Comput Biol. 2019 May 20;15(5):e1007037. doi: 10.1371/journal.pcbi.1007037 (PMC6544333; doi:10.1371/journal.pcbi.1007037)
Supplement: S1 Table — Number of taxon pairs with positive, negative and insignificant interactions for Pearson correlation and short timescale Granger causality models of the gut. (DOCX) [file pcbi.1007037.s003.docx]

**S1 Table. Correlation vs short timescale causality in the gut.** Number of taxon pairs with positive, negative and insignificant interactions for Pearson correlation and short timescale Granger causality models of the gut.

|  | Pearson | | | |
| --- | --- | --- | --- | --- |
| Granger |  | positive | negative | none |
|  | positive | 2 | 5 | 28 |
|  | negative | 21 | 0 | 27 |
|  | none | 19 | 6 | 145 |

Chi-square: 41.1220, *p*<0.00001
