# Supplementary material for: Detecting interaction networks in the human microbiome with conditional Granger causality
Source: PLoS Comput Biol. 2019 May 20;15(5):e1007037. doi: 10.1371/journal.pcbi.1007037 (PMC6544333; doi:10.1371/journal.pcbi.1007037)
Supplement: S2 Table — Number of taxon pairs with positive, negative and insignificant interactions for Pearson correlation and long timescale Granger causality models of the gut. (DOCX) [file pcbi.1007037.s004.docx]

**S2 Table. Correlation vs long timescale causality in the gut.** Number of taxon pairs with positive, negative and insignificant interactions for Pearson correlation and long timescale Granger causality models of the gut.

|  | Pearson | | | |
| --- | --- | --- | --- | --- |
| Granger |  | positive | negative | none |
|  | positive | 9 | 1 | 22 |
|  | negative | 8 | 0 | 16 |
|  | none | 25 | 10 | 162 |

Chi-square: 10.9638, *p* = 0.027
