# Supplementary material for: Detecting interaction networks in the human microbiome with conditional Granger causality
Source: PLoS Comput Biol. 2019 May 20;15(5):e1007037. doi: 10.1371/journal.pcbi.1007037 (PMC6544333; doi:10.1371/journal.pcbi.1007037)
Supplement: S3 Table — Number of taxon pairs with positive, negative and insignificant interactions for Pearson correlation and short timescale Granger causality models of the left-hand. (DOCX) [file pcbi.1007037.s005.docx]

**S3 Table. Correlation vs short timescale causality on the left-hand.** Number of taxon pairs with positive, negative and insignificant interactions for Pearson correlation and short timescale Granger causality models of the left-hand.

|  | Pearson | | | |
| --- | --- | --- | --- | --- |
| Granger |  | positive | negative | none |
|  | positive | 7 | 7 | 84 |
|  | negative | 38 | 3 | 68 |
|  | none | 62 | 20 | 414 |

Chi-square: 42.1073, *p* < 0.00001
