# Supplementary material for: Detecting interaction networks in the human microbiome with conditional Granger causality
Source: PLoS Comput Biol. 2019 May 20;15(5):e1007037. doi: 10.1371/journal.pcbi.1007037 (PMC6544333; doi:10.1371/journal.pcbi.1007037)
Supplement: S4 Table — Number of taxon pairs with positive, negative and insignificant interactions for Pearson correlation and long timescale Granger causality models of the left-hand. (DOCX) [file pcbi.1007037.s006.docx]

**S4 Table. Correlation vs long timescale causality on the left-hand.** Number of taxon pairs with positive, negative and insignificant interactions for Pearson correlation and long timescale Granger causality models of the left-hand.

|  | Pearson | | | |
| --- | --- | --- | --- | --- |
| Granger |  | positive | negative | none |
|  | positive | 23 | 5 | 78 |
|  | negative | 11 | 2 | 58 |
|  | none | 73 | 23 | 430 |

Chi-square: 4.7061, *p* = 0.32
