# Supplementary material for: Detecting interaction networks in the human microbiome with conditional Granger causality
Source: PLoS Comput Biol. 2019 May 20;15(5):e1007037. doi: 10.1371/journal.pcbi.1007037 (PMC6544333; doi:10.1371/journal.pcbi.1007037)
Supplement: S6 Table — Number of taxon pairs with positive, negative and insignificant interactions for Pearson correlation and long timescale Granger causality models of the right-hand. (DOCX) [file pcbi.1007037.s008.docx]

**S6 Table. Correlation vs long timescale causality on the right-hand.** Number of taxon pairs with positive, negative and insignificant interactions for Pearson correlation and long timescale Granger causality models of the right-hand.

|  | Pearson | | | |
| --- | --- | --- | --- | --- |
| Granger |  | positive | negative | none |
|  | positive | 19 | 2 | 172 |
|  | negative | 23 | 3 | 127 |
|  | none | 116 | 10 | 854 |

Chi-square: 3.3822, *p* = 0.50
