# Supplementary material for: Detecting interaction networks in the human microbiome with conditional Granger causality
Source: PLoS Comput Biol. 2019 May 20;15(5):e1007037. doi: 10.1371/journal.pcbi.1007037 (PMC6544333; doi:10.1371/journal.pcbi.1007037)
Supplement: S7 Table — Number of taxon pairs with positive, negative and insignificant interactions for Pearson correlation and short timescale Granger causality models of the tongue. (DOCX) [file pcbi.1007037.s009.docx]

**S7 Table.** **Correlation vs short timescale causality on the tongue.** Number of taxon pairs with positive, negative and insignificant interactions for Pearson correlation and short timescale Granger causality models of the tongue.

|  | Pearson | | | |
| --- | --- | --- | --- | --- |
| Granger |  | positive | negative | none |
|  | positive | 6 | 11 | 43 |
|  | negative | 38 | 1 | 26 |
|  | none | 51 | 34 | 196 |

Chi-square: 57.9082, *p*< 0.00001
