# Supplementary material for: Detecting interaction networks in the human microbiome with conditional Granger causality
Source: PLoS Comput Biol. 2019 May 20;15(5):e1007037. doi: 10.1371/journal.pcbi.1007037 (PMC6544333; doi:10.1371/journal.pcbi.1007037)
Supplement: S8 Table — Number of taxon pairs with positive, negative and insignificant interactions for Pearson correlation and long timescale Granger causality models of the tongue. (DOCX) [file pcbi.1007037.s010.docx]

**S8 Table. Correlation vs long timescale causality on the tongue.** Number of taxon pairs with positive, negative and insignificant interactions for Pearson correlation and long timescale Granger causality models of the tongue.

|  | Pearson | | | |
| --- | --- | --- | --- | --- |
| Granger |  | positive | negative | none |
|  | positive | 14 | 4 | 36 |
|  | negative | 15 | 2 | 31 |
|  | none | 66 | 40 | 198 |

Chi-square: 5.6064, *p* =0.23
