# Supplementary material for: Detecting interaction networks in the human microbiome with conditional Granger causality
Source: PLoS Comput Biol. 2019 May 20;15(5):e1007037. doi: 10.1371/journal.pcbi.1007037 (PMC6544333; doi:10.1371/journal.pcbi.1007037)
Supplement: S9 Table — (DOCX) [file pcbi.1007037.s011.docx]

**S9 Table. Comparison of Granger Causality Models Across Body Sites.**

| Sites | Taxon Interactions | | Average Number of Lag Coefficients | | | | |
| --- | --- | --- | --- | --- | --- | --- | --- |
|  | Possible | Actual | Total | Conserved  (no Sign Change) | Conserved  (Sign Change) | Sign Change | Not Conserved |
| Interspecific Interactions | | | | | | | |
| (4) Gut/L-Palm/R-Palm/Tongue | 0 | 0 | - | - | - | - | - |
| (3) Gut/L-Palm/R-Palm | 2 | 2 | 10.5 | 0 | 0 | 1.5 | 10.5 |
| (3) Gut/L-Palm/Tongue | 0 | 0 | - | - | - | - | - |
| (3) Gut/R-Palm/Tongue | 0 | 0 | - | - | - | - | - |
| (3) L-Palm/R-Palm/Tongue | 272 | 109 | 5.79 | 0.01 | 0.02 | 0.26 | 5.77 |
| (2) Gut/L-Palm | 2 | 2 | 9.00 | 0 | 0.50 | 0.50 | 8.50 |
| (2) Gut/R-Palm | 6 | 3 | 7.80 | 0.60 | 0.60 | 0.60 | 6.60 |
| (2) Gut/Tongue | 0 | 0 | - | - | - | - | - |
| (2) L-Palm/R-Palm | 1406 | 729 | 5.06 | 0.27 | 0.16 | 0.16 | 4.63 |
| (2) L-Palm/Tongue | 272 | 125 | 3.99 | 0.11 | 0.08 | 0.08 | 3.80 |
| (2) R-Palm/Tongue | 306 | 167 | 4.38 | 0.15 | 0.12 | 0.12 | 4.11 |
| (1) Gut | 506 | 360 | 4.86 | - | - | - | - |
| (1) L-Palm | 1406 | 968 | 3.83 | - | - | - | - |
| (1) R-Palm | 2652 | 1899 | 3.36 | - | - | - | - |
| (1) Tongue | 812 | 561 | 2.97 | - | - | - | - |
| Intraspecific Interactions | | | | | | | |
| (4) Gut/L-Palm/R-Palm/Tongue | 1 | 1 | 12.0 | 2.00 | 0 | 1.00 | 10.0 |
| (3) Gut/L-Palm/R-Palm | 2 | 2 | 11.5 | 1.50 | 0 | 1.00 | 10.0 |
| (3) Gut/L-Palm/Tongue | 1 | 1 | 11.0 | 2.00 | 0 | 0 | 9.00 |
| (3) Gut/R-Palm/Tongue | 1 | 1 | 11.0 | 2.00 | 0 | 1.00 | 9.00 |
| (3) L-Palm/R-Palm/Tongue | 17 | 17 | 8.29 | 1.59 | 0 | 0.29 | 6.71 |
| (2) Gut/L-Palm | 2 | 2 | 11.0 | 3.50 | 0.50 | 0.50 | 7.00 |
| (2) Gut/R-Palm | 3 | 3 | 8.00 | 2.00 | 0.33 | 0.33 | 5.66 |
| (2) Gut/Tongue | 1 | 1 | 10.0 | 3.00 | 0 | 0 | 7.00 |
| (2) L-Palm/R-Palm | 38 | 37 | 6.50 | 1.74 | 0.05 | 0.05 | 4.71 |
| (2) L-Palm/Tongue | 17 | 17 | 6.12 | 1.76 | 0.18 | 0.18 | 4.18 |
| (2) R-Palm/Tongue | 18 | 18 | 6.44 | 1.78 | 0.11 | 0.11 | 4.56 |
| (1) Gut | 23 | 20 | - | - | - | - | - |
| (1) L-Palm | 38 | 37 | - | - | - | - | - |
| (1) R-Palm | 52 | 51 | - | - | - | - | - |
| (1) Tongue | 29 | 29 | - | - | - | - | - |
