# Supplementary material for: Detecting interaction networks in the human microbiome with conditional Granger causality
Source: PLoS Comput Biol. 2019 May 20;15(5):e1007037. doi: 10.1371/journal.pcbi.1007037 (PMC6544333; doi:10.1371/journal.pcbi.1007037)
Supplement: S10 Table — (DOCX) [file pcbi.1007037.s012.docx]

**S10 Table. Conserved interactions including both interspecific (black) and intraspecific (red) interactions.**

| Sites | Effect | Cause | Lag (Positive) | Lag (Negative) |
| --- | --- | --- | --- | --- |
| (4) Gut/L-Palm/R-Palm/Tongue | Streptococcus | Streptococcus | - | 2,1 |
| (3) Gut/L-Palm/R-Palm | Bacteroides  Streptococcus | Bacteroides  Streptococcus | -  - | 1  2,1 |
| (3) Gut/L-Palm/Tongue | Streptococcus | Streptococcus | - | 2,1 |
| (3) Gut/R-Palm/Tongue | Streptococcus | Streptococcus | - | 2,1 |
| (3) L-Palm/R-Palm/Tongue | \| Aggregatibacter \| \| --- \| \| Fusobacterium \| \| Leptotrichia \| \| Granulicatella \| \| Neisseria \| \| Porphyromonas \| \| Campylobacter \| \| Gemella \| \| Lautropia \| \| Veillonella \| \| Corynebacterium \| \| Streptococcus \| \| Rothia \| \| Rothia \| \| Rothia \| \| Rothia \| \| Prevotella \| \| Actinomyces \| \| Haemophilus \| \| Capnocytophaga \| | \| Aggregatibacter \| \| --- \| \| Fusobacterium \| \| Leptotrichia \| \| Granulicatella \| \| Neisseria \| \| Porphyromonas \| \| Campylobacter \| \| Gemella \| \| Lautropia \| \| Veillonella \| \| Corynebacterium \| \| Streptococcus \| \| Neisseria \| \| Veillonella \| \| Corynebacterium \| \| Rothia \| \| Prevotella \| \| Actinomyces \| \| Haemophilus \| \| Capnocytophaga \| | \| - \| \| --- \| \| - \| \| - \| \| - \| \| - \| \| - \| \| - \| \| - \| \| - \| \| - \| \| - \| \| - \| \| - \| \| 17 \| \| 1 \| \| - \| \| - \| \| - \| \| - \| \| - \| | \| 2,1 \| \| --- \| \| 2,1 \| \| 1 \| \| 2,1 \| \| 2,1 \| \| 1 \| \| 1 \| \| 2,3,1 \| \| 1 \| \| 2,1 \| \| 2,1 \| \| 2,1 \| \| 2 \| \| - \| \| - \| \| 1 \| \| 2,1 \| \| 1 \| \| 1 \| \| 2 \| |
| (2) Gut/L-Palm | Bacteroides  Streptococcus | Bacteroides  Streptococcus | 8  - | 6,3,2,1  2,1 |
| (2) Gut/R-Palm | Bacteroides  Bacteroides  Clostridium  Streptococcus  Streptococcus  Streptococcus | Bacteroides  Streptococcus  Clostridium  Bacteroides  Clostridium  Streptococcus | -  11  -  3  -  - | 1  -  4,1  -  6  3,2,1 |
| (2) Gut/Tongue | Streptococcus | Streptococcus | 14 | 2,1 |
| (2) L-Palm/R-Palm | \| Acinetobacter \| \| --- \| \| Acinetobacter \| \| Hymenobacter \| \| Hymenobacter \| \| Hymenobacter \| \| Hymenobacter \| \| Hymenobacter \| \| Hymenobacter \| \| Hymenobacter \| \| Hymenobacter \| \| Facklamia \| \| Facklamia \| \| Peptoniphilus \| \| Peptoniphilus \| \| Peptoniphilus \| \| Peptoniphilus \| \| Peptoniphilus \| \| Peptoniphilus \| \| Peptoniphilus \| \| Peptoniphilus \| \| Peptoniphilus \| \| Peptoniphilus \| \| Peptoniphilus \| \| Peptoniphilus \| \| Peptoniphilus \| \| Peptoniphilus \| \| Peptoniphilus \| \| Peptoniphilus \| \| Aggregatibacter \| \| Aggregatibacter \| \| Aggregatibacter \| \| Aggregatibacter \| \| Aggregatibacter \| \| Aggregatibacter \| \| Aggregatibacter \| \| Aggregatibacter \| \| Aggregatibacter \| \| Aggregatibacter \| \| Paracoccus \| \| Paracoccus \| \| Paracoccus \| \| Paracoccus \| \| Paracoccus \| \| Paracoccus \| \| Paracoccus \| \| Paracoccus \| \| Paracoccus \| \| Paracoccus \| \| Paracoccus \| \| Paracoccus \| \| Paracoccus \| \| Paracoccus \| \| Paracoccus \| \| Paracoccus \| \| Paracoccus \| \| Paracoccus \| \| Paracoccus \| \| Paracoccus \| \| Paracoccus \| \| Paracoccus \| \| Paracoccus \| \| Finegoldia \| \| Finegoldia \| \| Finegoldia \| \| Finegoldia \| \| Finegoldia \| \| Finegoldia \| \| Fusobacterium \| \| Fusobacterium \| \| Fusobacterium \| \| Staphylococcus \| \| Leptotrichia \| \| Leptotrichia \| \| Leptotrichia \| \| Leptotrichia \| \| Leptotrichia \| \| Leptotrichia \| \| Leptotrichia \| \| Granulicatella \| \| Granulicatella \| \| Granulicatella \| \| Granulicatella \| \| Granulicatella \| \| Granulicatella \| \| Granulicatella \| \| Granulicatella \| \| Granulicatella \| \| Bacillus \| \| Bacillus \| \| Bacillus \| \| Bacillus \| \| Bacillus \| \| Bacillus \| \| Bacillus \| \| Bacillus \| \| Bacillus \| \| Bacillus \| \| Bacillus \| \| Bacillus \| \| Bacillus \| \| Bacillus \| \| Bacillus \| \| Bacillus \| \| Bacillus \| \| Bacillus \| \| Bacillus \| \| Bacillus \| \| Bacillus \| \| Bacillus \| \| Bacillus \| \| Bacillus \| \| Bacillus \| \| Bacillus \| \| Bacillus \| \| Brachybacterium \| \| Brachybacterium \| \| Brachybacterium \| \| Brachybacterium \| \| Brachybacterium \| \| Brachybacterium \| \| Brachybacterium \| \| Brachybacterium \| \| Brachybacterium \| \| Brachybacterium \| \| Brachybacterium \| \| Brachybacterium \| \| Brachybacterium \| \| Brachybacterium \| \| Brachybacterium \| \| Brachybacterium \| \| Brachybacterium \| \| Brachybacterium \| \| Brachybacterium \| \| Brachybacterium \| \| Brachybacterium \| \| Brachybacterium \| \| Neisseria \| \| Neisseria \| \| Neisseria \| \| Neisseria \| \| Neisseria \| \| Sphingomonas \| \| Sphingomonas \| \| Sphingomonas \| \| Sphingomonas \| \| Sphingomonas \| \| Sphingomonas \| \| Sphingomonas \| \| Sphingomonas \| \| Sphingomonas \| \| Sphingomonas \| \| Sphingomonas \| \| Sphingomonas \| \| Sphingomonas \| \| Sphingomonas \| \| Pseudomonas \| \| Pseudomonas \| \| Pseudomonas \| \| Porphyromonas \| \| Porphyromonas \| \| Porphyromonas \| \| Porphyromonas \| \| Porphyromonas \| \| Porphyromonas \| \| Porphyromonas \| \| Porphyromonas \| \| Bacteroides \| \| Campylobacter \| \| Gemella \| \| Gemella \| \| Gemella \| \| Gemella \| \| Gemella \| \| Gemella \| \| Gemella \| \| Gemella \| \| Gemella \| \| Gemella \| \| Brevibacterium \| \| Lautropia \| \| Lautropia \| \| Anaerococcus \| \| Anaerococcus \| \| Anaerococcus \| \| Anaerococcus \| \| Anaerococcus \| \| Anaerococcus \| \| Arthrobacter \| \| Arthrobacter \| \| Arthrobacter \| \| Arthrobacter \| \| Veillonella \| \| Veillonella \| \| Veillonella \| \| Veillonella \| \| Veillonella \| \| Veillonella \| \| Veillonella \| \| Veillonella \| \| Corynebacterium \| \| Corynebacterium \| \| Streptococcus \| \| Streptococcus \| \| Streptococcus \| \| Streptococcus \| \| Streptococcus \| \| Streptococcus \| \| Streptococcus \| \| Streptococcus \| \| Streptococcus \| \| Propionibacterium \| \| Propionibacterium \| \| Rothia \| \| Rothia \| \| Rothia \| \| Rothia \| \| Rothia \| \| Rothia \| \| Rothia \| \| Rothia \| \| Rothia \| \| Rothia \| \| Rothia \| \| Rothia \| \| Rothia \| \| Rothia \| \| Rothia \| \| Rothia \| \| Rothia \| \| Rothia \| \| Rothia \| \| Rothia \| \| Rothia \| \| Rothia \| \| Prevotella \| \| Prevotella \| \| Lactobacillus \| \| Lactococcus \| \| Actinomyces \| \| Actinomyces \| \| Actinomyces \| \| Actinomyces \| \| Micrococcus \| \| Micrococcus \| \| Micrococcus \| \| Micrococcus \| \| Micrococcus \| \| Micrococcus \| \| Micrococcus \| \| Micrococcus \| \| Micrococcus \| \| Micrococcus \| \| Micrococcus \| \| Micrococcus \| \| Micrococcus \| \| Micrococcus \| \| Micrococcus \| \| Micrococcus \| \| Micrococcus \| \| Micrococcus \| \| Micrococcus \| \| Haemophilus \| \| Haemophilus \| \| Haemophilus \| \| Haemophilus \| \| Haemophilus \| \| Haemophilus \| \| Haemophilus \| \| Haemophilus \| \| Haemophilus \| \| Haemophilus \| \| Haemophilus \| \| Methylobacterium \| \| Methylobacterium \| \| Methylobacterium \| \| Methylobacterium \| \| Methylobacterium \| \| Methylobacterium \| \| Methylobacterium \| \| Capnocytophaga \| \| Capnocytophaga \| \| Capnocytophaga \| \| Capnocytophaga \| \| Capnocytophaga \| | \| Acinetobacter \| \| --- \| \| Brachybacterium \| \| Acinetobacter \| \| Hymenobacter \| \| Brachybacterium \| \| Neisseria \| \| Streptococcus \| \| Propionibacterium \| \| Prevotella \| \| Methylobacterium \| \| Facklamia \| \| Prevotella \| \| Acinetobacter \| \| Facklamia \| \| Peptoniphilus \| \| Aggregatibacter \| \| Paracoccus \| \| Granulicatella \| \| Brachybacterium \| \| Neisseria \| \| Pseudomonas \| \| Porphyromonas \| \| Anaerococcus \| \| Arthrobacter \| \| Propionibacterium \| \| Lactobacillus \| \| Lactococcus \| \| Capnocytophaga \| \| Aggregatibacter \| \| Staphylococcus \| \| Granulicatella \| \| Porphyromonas \| \| Brevibacterium \| \| Arthrobacter \| \| Streptococcus \| \| Propionibacterium \| \| Prevotella \| \| Lactobacillus \| \| Acinetobacter \| \| Hymenobacter \| \| Aggregatibacter \| \| Paracoccus \| \| Staphylococcus \| \| Granulicatella \| \| Brachybacterium \| \| Sphingomonas \| \| Pseudomonas \| \| Porphyromonas \| \| Campylobacter \| \| Gemella \| \| Brevibacterium \| \| Lautropia \| \| Arthrobacter \| \| Veillonella \| \| Streptococcus \| \| Propionibacterium \| \| Prevotella \| \| Erwinia \| \| Lactococcus \| \| Micrococcus \| \| Haemophilus \| \| Facklamia \| \| Peptoniphilus \| \| Finegoldia \| \| Lactobacillus \| \| Lactococcus \| \| Haemophilus \| \| Fusobacterium \| \| Pseudomonas \| \| Lactococcus \| \| Micrococcus \| \| Leptotrichia \| \| Pseudomonas \| \| Campylobacter \| \| Brevibacterium \| \| Streptococcus \| \| Propionibacterium \| \| Erwinia \| \| Acinetobacter \| \| Granulicatella \| \| Neisseria \| \| Pseudomonas \| \| Campylobacter \| \| Streptococcus \| \| Rothia \| \| Prevotella \| \| Erwinia \| \| Acinetobacter \| \| Hymenobacter \| \| Facklamia \| \| Aggregatibacter \| \| Finegoldia \| \| Staphylococcus \| \| Leptotrichia \| \| Bacillus \| \| Brachybacterium \| \| Neisseria \| \| Sphingomonas \| \| Pseudomonas \| \| Bacteroides \| \| Gemella \| \| Brevibacterium \| \| Anaerococcus \| \| Arthrobacter \| \| Streptococcus \| \| Propionibacterium \| \| Rothia \| \| Prevotella \| \| Erwinia \| \| Lactobacillus \| \| Lactococcus \| \| Micrococcus \| \| Haemophilus \| \| Capnocytophaga \| \| Acinetobacter \| \| Hymenobacter \| \| Facklamia \| \| Staphylococcus \| \| Bacillus \| \| Brachybacterium \| \| Neisseria \| \| Sphingomonas \| \| Pseudomonas \| \| Porphyromonas \| \| Gemella \| \| Brevibacterium \| \| Lautropia \| \| Anaerococcus \| \| Veillonella \| \| Streptococcus \| \| Propionibacterium \| \| Rothia \| \| Erwinia \| \| Lactococcus \| \| Actinomyces \| \| Micrococcus \| \| Acinetobacter \| \| Paracoccus \| \| Neisseria \| \| Propionibacterium \| \| Lactobacillus \| \| Facklamia \| \| Paracoccus \| \| Fusobacterium \| \| Brachybacterium \| \| Sphingomonas \| \| Porphyromonas \| \| Campylobacter \| \| Anaerococcus \| \| Propionibacterium \| \| Prevotella \| \| Erwinia \| \| Lactobacillus \| \| Micrococcus \| \| Haemophilus \| \| Pseudomonas \| \| Propionibacterium \| \| Lactococcus \| \| Staphylococcus \| \| Leptotrichia \| \| Brachybacterium \| \| Porphyromonas \| \| Campylobacter \| \| Brevibacterium \| \| Streptococcus \| \| Lactobacillus \| \| Bacteroides \| \| Campylobacter \| \| Acinetobacter \| \| Facklamia \| \| Finegoldia \| \| Granulicatella \| \| Brachybacterium \| \| Pseudomonas \| \| Porphyromonas \| \| Gemella \| \| Brevibacterium \| \| Arthrobacter \| \| Brevibacterium \| \| Brevibacterium \| \| Lautropia \| \| Peptoniphilus \| \| Pseudomonas \| \| Anaerococcus \| \| Streptococcus \| \| Lactobacillus \| \| Lactococcus \| \| Lautropia \| \| Arthrobacter \| \| Veillonella \| \| Erwinia \| \| Acinetobacter \| \| Paracoccus \| \| Leptotrichia \| \| Veillonella \| \| Propionibacterium \| \| Rothia \| \| Prevotella \| \| Haemophilus \| \| Paracoccus \| \| Corynebacterium \| \| Acinetobacter \| \| Paracoccus \| \| Finegoldia \| \| Brachybacterium \| \| Gemella \| \| Corynebacterium \| \| Streptococcus \| \| Rothia \| \| Micrococcus \| \| Propionibacterium \| \| Lactococcus \| \| Acinetobacter \| \| Peptoniphilus \| \| Aggregatibacter \| \| Paracoccus \| \| Staphylococcus \| \| Granulicatella \| \| Neisseria \| \| Sphingomonas \| \| Campylobacter \| \| Lautropia \| \| Anaerococcus \| \| Arthrobacter \| \| Veillonella \| \| Corynebacterium \| \| Propionibacterium \| \| Rothia \| \| Prevotella \| \| Erwinia \| \| Lactobacillus \| \| Lactococcus \| \| Micrococcus \| \| Methylobacterium \| \| Prevotella \| \| Micrococcus \| \| Lactobacillus \| \| Lactococcus \| \| Facklamia \| \| Fusobacterium \| \| Lactobacillus \| \| Actinomyces \| \| Aggregatibacter \| \| Paracoccus \| \| Granulicatella \| \| Brachybacterium \| \| Neisseria \| \| Porphyromonas \| \| Bacteroides \| \| Brevibacterium \| \| Arthrobacter \| \| Veillonella \| \| Streptococcus \| \| Rothia \| \| Prevotella \| \| Erwinia \| \| Lactobacillus \| \| Lactococcus \| \| Micrococcus \| \| Haemophilus \| \| Methylobacterium \| \| Acinetobacter \| \| Neisseria \| \| Porphyromonas \| \| Bacteroides \| \| Gemella \| \| Anaerococcus \| \| Arthrobacter \| \| Prevotella \| \| Erwinia \| \| Lactococcus \| \| Haemophilus \| \| Peptoniphilus \| \| Porphyromonas \| \| Propionibacterium \| \| Prevotella \| \| Erwinia \| \| Micrococcus \| \| Methylobacterium \| \| Leptotrichia \| \| Brevibacterium \| \| Prevotella \| \| Lactococcus \| \| Capnocytophaga \| | \| \| - \| \| \| --- \| --- \| \| 13 \| \| \| - \| \| \| - \| \| \| - \| \| \| - \| \| \| 14 \| \| \| - \| \| \| 7 \| \| \| - \| \| \| - \| \| \| 14 \| \| \| 10 \| \| \| 18 \| \| \| - \| \| \| - \| \| \| 20 \| \| \| - \| \| \| - \| \| \| 1 \| \| \| 8 \| \| \| 3,18,15 \| \| \| - \| \| \| - \| \| \| 2 \| \| \| - \| \| \| 6 \| \| \| 5 \| \| \| - \| \| \| 8 \| \| \| 19 \| \| \| - \| \| \| 11 \| \| \| 1 \| \| \| - \| \| \| 14 \| \| \| - \| \| \| 16 \| \| \| 9 \| \| \| - \| \| \| - \| \| \| - \| \| \| - \| \| \| - \| \| \| - \| \| \| - \| \| \| 5 \| \| \| 18 \| \| \| 3 \| \| \| 9 \| \| \| 16,14 \| \| \| - \| \| \| - \| \| \| 3,20 \| \| \| 7,14 \| \| \| - \| \| \| 7 \| \| \| 2,18 \| \| \| 16 \| \| \| - \| \| \| 20 \| \| \| - \| \| \| - \| \| \| - \| \| \| - \| \| \| 11 \| \| \| 9 \| \| \| - \| \| \| 7 \| \| \| 8 \| \| \| 1 \| \| \| - \| \| \| 7 \| \| \| - \| \| \| 2 \| \| \| 17 \| \| \| 15 \| \| \| 2 \| \| \| - \| \| \| - \| \| \| 7 \| \| \| 1 \| \| \| 13 \| \| \| - \| \| \| 16 \| \| \| - \| \| \| 2 \| \| \| 17,15,3 \| \| \| 12,4 \| \| \| 10 \| \| \| 16,9,6 \| \| \| 2 \| \| \| - \| \| \| - \| \| \| - \| \| \| 2 \| \| \| - \| \| \| - \| \| \| 2 \| \| \| 1 \| \| \| 19 \| \| \| - \| \| \| 15 \| \| \| 10 \| \| \| 14,3 \| \| \| 7 \| \| \| 12 \| \| \| 14 \| \| \| 10,6 \| \| \| 20 \| \| \| - \| \| \| 2 \| \| \| 14 \| \| \| - \| \| \| 15 \| \| \| - \| \| \| 6 \| \| \| 6 \| \| \| - \| \| \| - \| \| \| - \| \| \| - \| \| \| 4 \| \| \| 15 \| \| \| 14 \| \| \| 19 \| \| \| - \| \| \| 5 \| \| \| - \| \| \| 14 \| \| \| - \| \| \| 15 \| \| \| 18,15,4 \| \| \| 5 \| \| \| 20 \| \| \| - \| \| \| 11 \| \| \| - \| \| \| - \| \| \| 14 \| \| \| 18 \| \| \| 17 \| \| \| - \| \| \| 1 \| \| \| - \| \| \| - \| \| \| - \| \| \| 6 \| \| \| - \| \| \| - \| \| \| - \| \| \| 18,10,4 \| \| \| 8 \| \| \| 11 \| \| \| - \| \| \| - \| \| \| 16 \| \| \| - \| \| \| 6 \| \| \| 8 \| \| \| - \| \| \| - \| \| 16,6 \| \| \| 3 \| \| \| 1 \| \| \| 8 \| \| \| - \| \| \| - \| \| \| 11 \| \| \| 20 \| \| \| 12 \| \| \| 19 \| \| \| 12 \| \| \| 3 \| \| \| - \| \| \| - \| \| \| 11 \| \| \| 6 \| \| \| - \| \| \| 11 \| \| \| - \| \| \| - \| \| \| - \| \| \| - \| \| \| - \| \| \| 5 \| \| \| 2 \| \| \| 1 \| \| \| - \| \| \| 3 \| \| \| 10 \| \| \| 11 \| \| \| - \| \| \| - \| \| \| - \| \| \| 9 \| \| \| 16 \| \| \| 16 \| \| \| 18 \| \| \| 4 \| \| \| - \| \| \| 11 \| \| \| - \| \| \| 2 \| \| \| 11 \| \| \| - \| \| \| 12 \| \| \| - \| \| \| 16 \| \| \| 5 \| \| \| - \| \| \| - \| \| \| 8,11 \| \| \| 13 \| \| \| - \| \| \| - \| \| \| 8,1 \| \| \| 19,11 \| \| \| - \| \| \| - \| \| \| 5,9 \| \| \| 19,10 \| \| \| 2 \| \| \| 1 \| \| \| 17 \| \| \| 1 \| \| \| 14 \| \| \| - \| \| \| 16 \| \| \| 12,10 \| \| \| - \| \| \| 15,10,2 \| \| \| 9 \| \| \| 16 \| \| \| - \| \| \| 10 \| \| \| - \| \| \| - \| \| \| 15 \| \| \| - \| \| \| - \| \| \| - \| \| \| 16 \| \| \| 8 \| \| \| 17 \| \| \| - \| \| \| 14 \| \| \| 2,4 \| \| \| - \| \| \| 19 \| \| \| 3 \| \| \| 20,10 \| \| \| 7 \| \| \| 13 \| \| \| - \| \| \| - \| \| \| 5 \| \| \| 10,6 \| \| \| - \| \| \| 3 \| \| \| - \| \| \| 11 \| \| \| - \| \| \| 4 \| \| \| 18 \| \| \| - \| \| \| 2 \| \| \| 6 \| \| \| - \| \| \| 12 \| \| \| - \| \| \| - \| \| \| - \| \| \| - \| \| \| - \| \| \| - \| \| \| 10 \| \| \| - \| \| \| - \| \| \| 16 \| \| \| 11 \| \| \| - \| \| \| 8 \| \| \| - \| \| \| \| --- \| --- \| --- \| --- \| --- \| --- \| --- \| --- \| --- \| --- \| --- \| --- \| --- \| --- \| --- \| --- \| --- \| --- \| --- \| --- \| --- \| --- \| --- \| --- \| --- \| --- \| --- \| --- \| --- \| --- \| --- \| --- \| --- \| --- \| --- \| --- \| --- \| --- \| --- \| --- \| --- \| --- \| --- \| --- \| --- \| --- \| --- \| --- \| --- \| --- \| --- \| --- \| --- \| --- \| --- \| --- \| --- \| --- \| --- \| --- \| --- \| --- \| --- \| --- \| --- \| --- \| --- \| --- \| --- \| --- \| --- \| --- \| --- \| --- \| --- \| --- \| --- \| --- \| --- \| --- \| --- \| --- \| --- \| --- \| --- \| --- \| --- \| --- \| --- \| --- \| --- \| --- \| --- \| --- \| --- \| --- \| --- \| --- \| --- \| --- \| --- \| --- \| --- \| --- \| --- \| --- \| --- \| --- \| --- \| --- \| --- \| --- \| --- \| --- \| --- \| --- \| --- \| --- \| --- \| --- \| --- \| --- \| --- \| --- \| --- \| --- \| --- \| --- \| --- \| --- \| --- \| --- \| --- \| --- \| --- \| --- \| --- \| --- \| --- \| --- \| --- \| --- \| --- \| --- \| --- \| --- \| --- \| --- \| --- \| --- \| --- \| --- \| --- \| --- \| --- \| --- \| --- \| --- \| --- \| --- \| --- \| --- \| --- \| --- \| --- \| --- \| --- \| --- \| --- \| --- \| --- \| --- \| --- \| --- \| --- \| --- \| --- \| --- \| --- \| --- \| --- \| --- \| --- \| --- \| --- \| --- \| --- \| --- \| --- \| --- \| --- \| --- \| --- \| --- \| --- \| --- \| --- \| --- \| --- \| --- \| --- \| --- \| --- \| --- \| --- \| --- \| --- \| --- \| --- \| --- \| --- \| --- \| --- \| --- \| --- \| --- \| --- \| --- \| --- \| --- \| --- \| --- \| --- \| --- \| --- \| --- \| --- \| --- \| --- \| --- \| --- \| --- \| --- \| --- \| --- \| --- \| --- \| --- \| --- \| --- \| --- \| --- \| --- \| --- \| --- \| --- \| --- \| --- \| --- \| --- \| --- \| --- \| --- \| --- \| --- \| --- \| --- \| --- \| --- \| --- \| --- \| --- \| --- \| --- \| --- \| --- \| --- \| --- \| --- \| --- \| --- \| --- \| --- \| --- \| --- \| --- \| --- \| --- \| --- \| --- \| --- \| --- \| --- \| --- \| --- \| --- \| --- \| --- \| --- \| --- \| --- \| --- \| --- \| --- \| --- \| --- \| --- \| --- \| --- \| --- \| --- \| --- \| --- \| --- \| --- \| --- \| --- \| --- \| --- \| --- \| --- \| --- \| --- \| --- \| --- \| --- \| --- \| --- \| --- \| --- \| --- \| --- \| --- \| --- \| --- \| --- \| --- \| --- \| --- \| --- \| --- \| --- \| --- \| --- \| --- \| --- \| --- \| --- \| --- \| --- \| --- \| --- \| --- \| --- \| --- \| --- \| --- \| --- \| --- \| --- \| --- \| --- \| --- \| --- \| --- \| --- \| --- \| --- \| --- \| --- \| --- \| --- \| --- \| --- \| --- \| --- \| --- \| --- \| --- \| --- \| --- \| --- \| --- \| --- \| --- \| --- \| --- \| --- \| --- \| --- \| --- \| --- \| --- \| --- \| --- \| --- \| --- \| --- \| --- \| --- \| --- \| --- \| --- \| --- \| --- \| --- \| --- \| --- \| --- \| --- \| --- \| --- \| --- \| --- \| --- \| --- \| --- \| --- \| --- \| --- \| --- \| --- \| --- \| --- \| --- \| --- \| --- \| --- \| --- \| --- \| --- \| --- \| --- \| --- \| --- \| --- \| --- \| --- \| --- \| --- \| --- \| --- \| --- \| --- \| --- \| --- \| --- \| --- \| --- \| --- \| --- \| --- \| --- \| --- \| --- \| --- \| --- \| --- \| --- \| --- \| --- \| --- \| --- \| --- \| --- \| --- \| --- \| --- \| --- \| --- \| --- \| --- \| --- \| --- \| --- \| --- \| --- \| --- \| --- \| --- \| --- \| --- \| --- \| --- \| --- \| --- \| --- \| --- \| --- \| --- \| --- \| --- \| --- \| --- \| --- \| --- \| --- \| --- \| --- \| --- \| --- \| --- \| --- \| --- \| --- \| --- \| --- \| --- \| --- \| --- \| --- \| --- \| --- \| --- \| --- \| --- \| --- \| --- \| --- \| --- \| --- \| --- \| --- \| --- \| --- \| --- \| --- \| --- \| --- \| --- \| --- \| --- \| --- \| --- \| --- \| --- \| --- \| --- \| --- \| --- \| --- \| --- \| --- \| --- \| --- \| --- \| --- \| --- \| --- \| --- \| --- \| --- \| --- \| --- \| --- \| --- \| --- \| --- \| --- \| --- \| --- \| --- \| --- \| --- \| --- \| --- \| --- \| --- \| --- \| --- \| --- \| --- \| --- \| --- \| --- \| --- \| --- \| --- \| | \| 1 \| \| --- \| \| - \| \| 1 \| \| 2,1 \| \| 2 \| \| 5 \| \| - \| \| 2 \| \| - \| \| 1 \| \| 2,1 \| \| - \| \| - \| \| - \| \| 2 \| \| 20 \| \| - \| \| 18 \| \| 18,14 \| \| - \| \| - \| \| - \| \| 3 \| \| 2 \| \| - \| \| 1 \| \| - \| \| - \| \| 2,1 \| \| - \| \| 20 \| \| 17 \| \| - \| \| - \| \| 18 \| \| - \| \| 2 \| \| 19 \| \| 1 \| \| 18 \| \| 6 \| \| 2,1 \| \| 2,8 \| \| 15 \| \| 15 \| \| 8 \| \| - \| \| - \| \| 7 \| \| - \| \| 12,18 \| \| 13 \| \| 2,5,11 \| \| - \| \| - \| \| 2,6 \| \| - \| \| 8 \| \| - \| \| 12 \| \| - \| \| 10 \| \| 2 \| \| 2,1 \| \| 1 \| \| - \| \| - \| \| 2,1 \| \| - \| \| - \| \| - \| \| 1 \| \| - \| \| 12 \| \| - \| \| - \| \| - \| \| - \| \| 20 \| \| 2,1 \| \| - \| \| - \| \| - \| \| 3,14 \| \| - \| \| 5 \| \| - \| \| 4 \| \| 1 \| \| 19,17,1 \| \| 11 \| \| - \| \| 6 \| \| 20 \| \| 3,2 \| \| 20 \| \| 20,5 \| \| 17 \| \| - \| \| - \| \| 17 \| \| 5 \| \| - \| \| 3,15 \| \| - \| \| - \| \| 1 \| \| - \| \| - \| \| 8 \| \| 20,3 \| \| - \| \| - \| \| 17,11,2 \| \| - \| \| 7 \| \| 1 \| \| - \| \| 3 \| \| 4,2,1 \| \| 5 \| \| 6 \| \| - \| \| 12,8 \| \| - \| \| 6 \| \| 2 \| \| 7 \| \| 9 \| \| - \| \| 7 \| \| 10 \| \| - \| \| 1 \| \| - \| \| 2 \| \| - \| \| 13 \| \| 2,1 \| \| - \| \| - \| \| - \| \| 4 \| \| - \| \| 6 \| \| 2,1 \| \| 15 \| \| - \| \| 7 \| \| 6 \| \| 12 \| \| - \| \| - \| \| - \| \| 8 \| \| 2,1 \| \| - \| \| 6 \| \| - \| \| - \| \| 2 \| \| 2,1 \| \| - \| \| - \| \| - \| \| - \| \| 1 \| \| 1 \| \| - \| \| - \| \| - \| \| - \| \| 15 \| \| - \| \| 5 \| \| 3,2,1 \| \| - \| \| - \| \| 1 \| \| - \| \| 1 \| \| 2 \| \| 11 \| \| 3,2,1 \| \| 8 \| \| - \| \| - \| \| - \| \| 3,1 \| \| - \| \| - \| \| - \| \| 18 \| \| 12 \| \| 2,1 \| \| - \| \| - \| \| - \| \| 1 \| \| - \| \| 2,1 \| \| - \| \| 13 \| \| - \| \| 15 \| \| 2 \| \| - \| \| 2,1 \| \| - \| \| - \| \| 2,1 \| \| 5 \| \| 18 \| \| - \| \| 20,15,1 \| \| 16 \| \| 11 \| \| 1 \| \| 2 \| \| 4 \| \| - \| \| - \| \| - \| \| - \| \| - \| \| - \| \| 5 \| \| 1,4 \| \| - \| \| 6 \| \| 19,1 \| \| 12 \| \| 8 \| \| - \| \| 2,1 \| \| - \| \| 2,1 \| \| 2,1 \| \| - \| \| 12 \| \| 4 \| \| 1 \| \| 9 \| \| 20,9 \| \| - \| \| 10 \| \| 15 \| \| 3,17 \| \| 7 \| \| 18,3,1 \| \| - \| \| - \| \| 17,8,6 \| \| - \| \| 4 \| \| 12 \| \| 9,3 \| \| 7 \| \| 3,2,1 \| \| - \| \| 20 \| \| - \| \| 1 \| \| 1 \| \| - \| \| 10 \| \| - \| \| - \| \| 2 \| \| - \| \| 11 \| \| 1 \| \| 10 \| \| 5 \| \| 2 \| \| 12 \| \| - \| \| 1 \| \| 2,1 \| \| 1 \| \| - \| \| 2 \| \| - \| \| 2 \| |
| (2) L-Palm/Tongue | \| Aggregatibacter \| \| --- \| \| Aggregatibacter \| \| Aggregatibacter \| \| Fusobacterium \| \| Leptotrichia \| \| Leptotrichia \| \| Leptotrichia \| \| Granulicatella \| \| Granulicatella \| \| Neisseria \| \| Neisseria \| \| Porphyromonas \| \| Campylobacter \| \| Gemella \| \| Lautropia \| \| Veillonella \| \| Veillonella \| \| Veillonella \| \| Veillonella \| \| Veillonella \| \| Veillonella \| \| Veillonella \| \| Corynebacterium \| \| Streptococcus \| \| Streptococcus \| \| Rothia \| \| Rothia \| \| Rothia \| \| Rothia \| \| Rothia \| \| Rothia \| \| Prevotella \| \| Prevotella \| \| Actinomyces \| \| Actinomyces \| \| Actinomyces \| \| Haemophilus \| \| Haemophilus \| \| Capnocytophaga \| | \| Aggregatibacter \| \| --- \| \| Granulicatella \| \| Campylobacter \| \| Fusobacterium \| \| Leptotrichia \| \| Corynebacterium \| \| Prevotella \| \| Granulicatella \| \| Porphyromonas \| \| Leptotrichia \| \| Neisseria \| \| Porphyromonas \| \| Campylobacter \| \| Gemella \| \| Lautropia \| \| Neisseria \| \| Campylobacter \| \| Gemella \| \| Veillonella \| \| Corynebacterium \| \| Prevotella \| \| Capnocytophaga \| \| Corynebacterium \| \| Neisseria \| \| Streptococcus \| \| Neisseria \| \| Gemella \| \| Veillonella \| \| Corynebacterium \| \| Streptococcus \| \| Rothia \| \| Campylobacter \| \| Prevotella \| \| Leptotrichia \| \| Campylobacter \| \| Actinomyces \| \| Aggregatibacter \| \| Haemophilus \| \| Capnocytophaga \| | \| - \| \| --- \| \| - \| \| - \| \| - \| \| - \| \| 19 \| \| - \| \| - \| \| - \| \| - \| \| - \| \| - \| \| - \| \| - \| \| - \| \| - \| \| - \| \| 8 \| \| - \| \| 19 \| \| 18 \| \| 20 \| \| - \| \| 16 \| \| - \| \| 11 \| \| 19 \| \| 17 \| \| 1 \| \| - \| \| - \| \| 6 \| \| - \| \| - \| \| - \| \| - \| \| 18 \| \| - \| \| - \| | \| 2,1 \| \| --- \| \| 1 \| \| 2 \| \| 2,1 \| \| 2,1 \| \| - \| \| 6 \| \| 3,2,1 \| \| 10 \| \| 13 \| \| 2,1 \| \| 1 \| \| 1 \| \| 2,3,1 \| \| 1 \| \| 1 \| \| 1 \| \| - \| \| 2,1 \| \| - \| \| - \| \| 8 \| \| 3,2,1 \| \| - \| \| 2,1 \| \| 2,20 \| \| 12 \| \| - \| \| - \| \| 8 \| \| 1 \| \| - \| \| 2,1 \| \| 4 \| \| 1 \| \| 1 \| \| - \| \| 1 \| \| 2 \| |
| (2) R-Palm/Tongue | \| Aggregatibacter \| \| --- \| \| Aggregatibacter \| \| Fusobacterium \| \| Fusobacterium \| \| Fusobacterium \| \| Leptotrichia \| \| Leptotrichia \| \| Leptotrichia \| \| Leptotrichia \| \| Leptotrichia \| \| Granulicatella \| \| Neisseria \| \| Porphyromonas \| \| Campylobacter \| \| Gemella \| \| Gemella \| \| Lautropia \| \| Lautropia \| \| Lautropia \| \| Lautropia \| \| Lautropia \| \| Lautropia \| \| Prevotella \| \| Corynebacterium \| \| Streptococcus \| \| Streptococcus \| \| Streptococcus \| \| Rothia \| \| Rothia \| \| Rothia \| \| Rothia \| \| Rothia \| \| Rothia \| \| Rothia \| \| Rothia \| \| Rothia \| \| Haemophilus \| \| Haemophilus \| \| Haemophilus \| \| Haemophilus \| \| Parvimonas \| \| Parvimonas \| \| Veillonella \| \| Veillonella \| \| Veillonella \| \| Veillonella \| \| Actinomyces \| \| Capnocytophaga \| \| Capnocytophaga \| \| Capnocytophaga \| \| Capnocytophaga \| \| Capnocytophaga \| | \| Aggregatibacter \| \| --- \| \| Granulicatella \| \| Fusobacterium \| \| Parvimonas \| \| Capnocytophaga \| \| Leptotrichia \| \| Granulicatella \| \| Campylobacter \| \| Prevotella \| \| Rothia \| \| Granulicatella \| \| Neisseria \| \| Porphyromonas \| \| Campylobacter \| \| Gemella \| \| Rothia \| \| Gemella \| \| Lautropia \| \| Corynebacterium \| \| Streptococcus \| \| Veillonella \| \| Capnocytophaga \| \| Prevotella \| \| Corynebacterium \| \| Granulicatella \| \| Streptococcus \| \| Rothia \| \| Neisseria \| \| Porphyromonas \| \| Campylobacter \| \| Gemella \| \| Lautropia \| \| Corynebacterium \| \| Rothia \| \| Parvimonas \| \| Veillonella \| \| Neisseria \| \| Rothia \| \| Haemophilus \| \| Capnocytophaga \| \| Campylobacter \| \| Parvimonas \| \| Fusobacterium \| \| Neisseria \| \| Rothia \| \| Veillonella \| \| Actinomyces \| \| Granulicatella \| \| Campylobacter \| \| Prevotella \| \| Rothia \| \| Capnocytophaga \| | \| - \| \| \| --- \| --- \| \| - \| \| \| - \| \| - \| \| \| - \| \| \| - \| \| \| 2 \| \| \| - \| \| \| 4 \| \| \| 16 \| \| \| - \| \| \| - \| \| \| - \| \| \| - \| \| \| - \| \| \| - \| \| \| 1 \| \| \| - \| \| \| 11 \| \| \| 14 \| \| \| 8 \| \| \| - \| \| \| - \| \| \| - \| \| \| - \| \| \| - \| \| \| 13 \| \| \| - \| \| \| 4,15 \| \| \| 14 \| \| \| 17 \| \| \| - \| \| \| 5,1 \| \| \| - \| \| \| - \| \| \| 17 \| \| \| - \| \| \| 16 \| \| \| - \| \| \| - \| \| \| 5 \| \| \| - \| \| \| 10 \| \| \| - \| \| \| 11 \| \| \| - \| \| \| - \| \| \| - \| \| \| - \| \| \| - \| \| \| 16 \| \| \| - \| \| | \| 2,1 \| \| --- \| \| 3 \| \| 2,1 \| \| 7 \| \| 3 \| \| 12,1 \| \| 17 \| \| 10 \| \| - \| \| 10 \| \| 2,1 \| \| 2,1 \| \| 1 \| \| 1 \| \| 3,2,1 \| \| 6 \| \| - \| \| 1 \| \| - \| \| - \| \| - \| \| 13 \| \| 2,1 \| \| 2,1 \| \| 13 \| \| 2,1 \| \| - \| \| 2 \| \| - \| \| 6 \| \| 14 \| \| 15 \| \| - \| \| 3,1 \| \| 15 \| \| - \| \| 3 \| \| - \| \| 1 \| \| 3 \| \| - \| \| 1 \| \| - \| \| 3 \| \| - \| \| 2,1 \| \| 1 \| \| 20 \| \| 2 \| \| 11 \| \| - \| \| 3,2,1 \| |
