# Supplementary material for: Detecting interaction networks in the human microbiome with conditional Granger causality
Source: PLoS Comput Biol. 2019 May 20;15(5):e1007037. doi: 10.1371/journal.pcbi.1007037 (PMC6544333; doi:10.1371/journal.pcbi.1007037)
Supplement: S11 Table — (DOCX) [file pcbi.1007037.s013.docx]

**S11 Table.** **Qualitative interactions conserved across the tongue, left- and right-hand.**

| Effect | Cause | Timescale | Sign |
| --- | --- | --- | --- |
| *Actinomyces* | *Campylobacter* | short | negative |
| *Actinomyces* | *Leptotrichia* | short | negative |
| *Aggregatibacter* | *Granulicatella* | short | negative |
| *Capnocytophaga* | *Fusobacterium* | short | negative |
| *Haemophilus* | *Neisseria* | short | negative |
| *Leptotrichia* | *Capnocytophaga* | short | negative |
| *Neisseria* | *Rothia* | short | negative |
| *Prevotella* | *Granulicatella* | short | negative |
| *Rothia* | *Neisseria* | short | negative |
| *Veillonella* | *Neisseria* | short | negative |
| *Leptotrichia* | *Actinomyces* | long | positive |
| *Prevotella* | *Porphyromonas* | long | positive |
