# Supplementary material for: Detecting interaction networks in the human microbiome with conditional Granger causality
Source: PLoS Comput Biol. 2019 May 20;15(5):e1007037. doi: 10.1371/journal.pcbi.1007037 (PMC6544333; doi:10.1371/journal.pcbi.1007037)
Supplement: S12 Table — Number of strong coefficients detected from randomized data as well as from real data. (DOCX) [file pcbi.1007037.s014.docx]

**S12 Table. Validating model with randomized data.** Number of strong coefficients detected from randomized data as well as from real data.

| Body site | Taxa Interaction | Number of significant taxa-pairs in original analysis (A) | Number of significant taxa-pairs in analysis of randomized data (B) | Number of significant taxa-pairs in both analyses | Ratio of B to A |
| --- | --- | --- | --- | --- | --- |
| Gut | positive | 38 | 3 | 2 | 0.08 |
| Gut | negative | 32 | 5 | 0 | 0.16 |
| Left Palm | positive | 89 | 4 | 1 | 0.04 |
| Left Palm | negative | 76 | 2 | 1 | 0.03 |
| Right Palm | positive | 147 | 9 | 1 | 0.06 |
| Right Palm | negative | 121 | 14 | 0 | 0.12 |
| Tongue | positive | 39 | 4 | 0 | 0.1 |
| Tongue | negative | 35 | 0 | 0 | 0 |
|  |  |  |  |  | **Average = 0.07** |
