# Supplementary figures and images for: Detecting interaction networks in the human microbiome with conditional Granger causality
Source: PLoS Comput Biol. 2019 May 20;15(5):e1007037. doi: 10.1371/journal.pcbi.1007037 (PMC6544333; doi:10.1371/journal.pcbi.1007037)

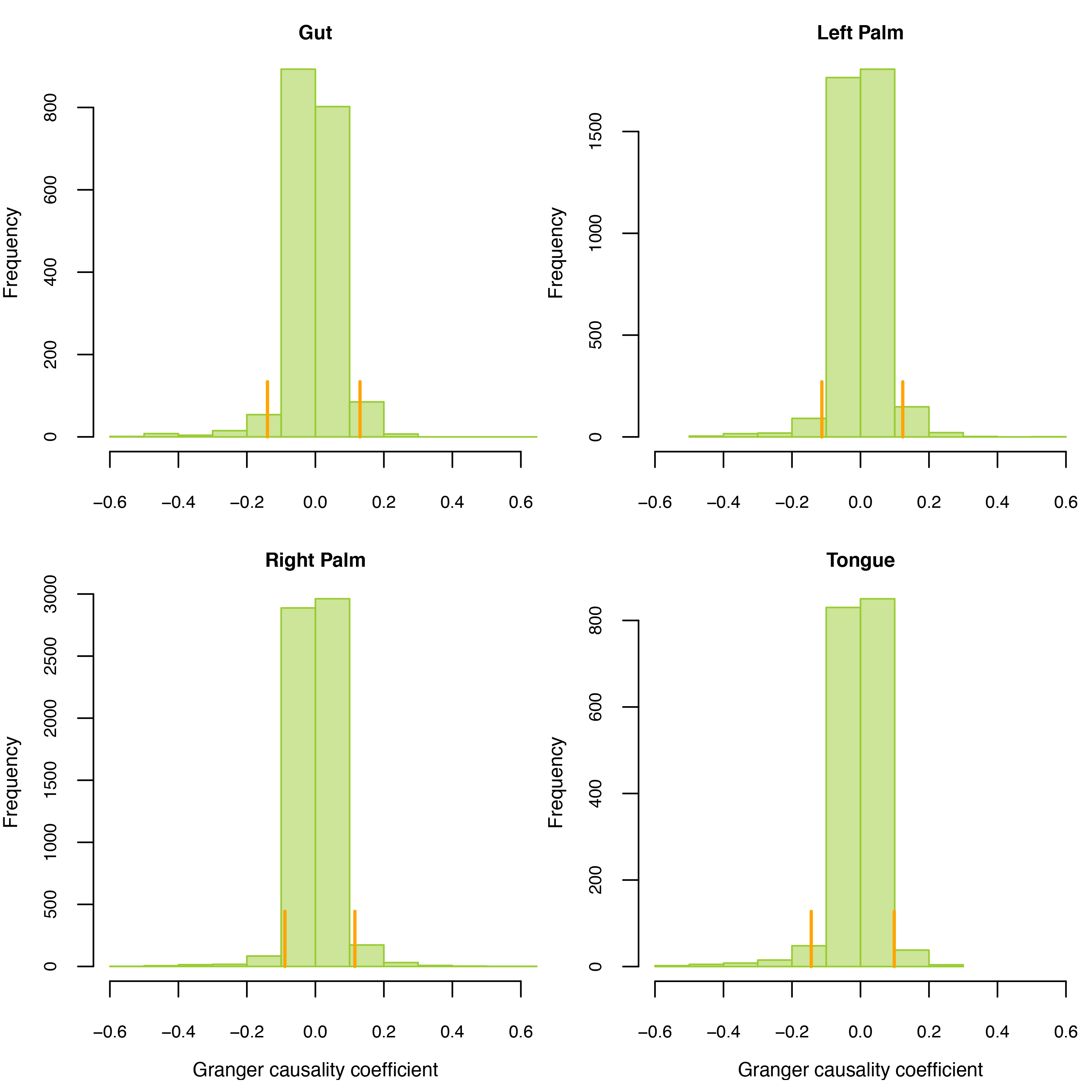

Supplement: S1 Fig — Effects outside of the orange bars correspond to the strongest 5% of effects in positive and negative interactions. (TIF) [file pcbi.1007037.s015.tif]

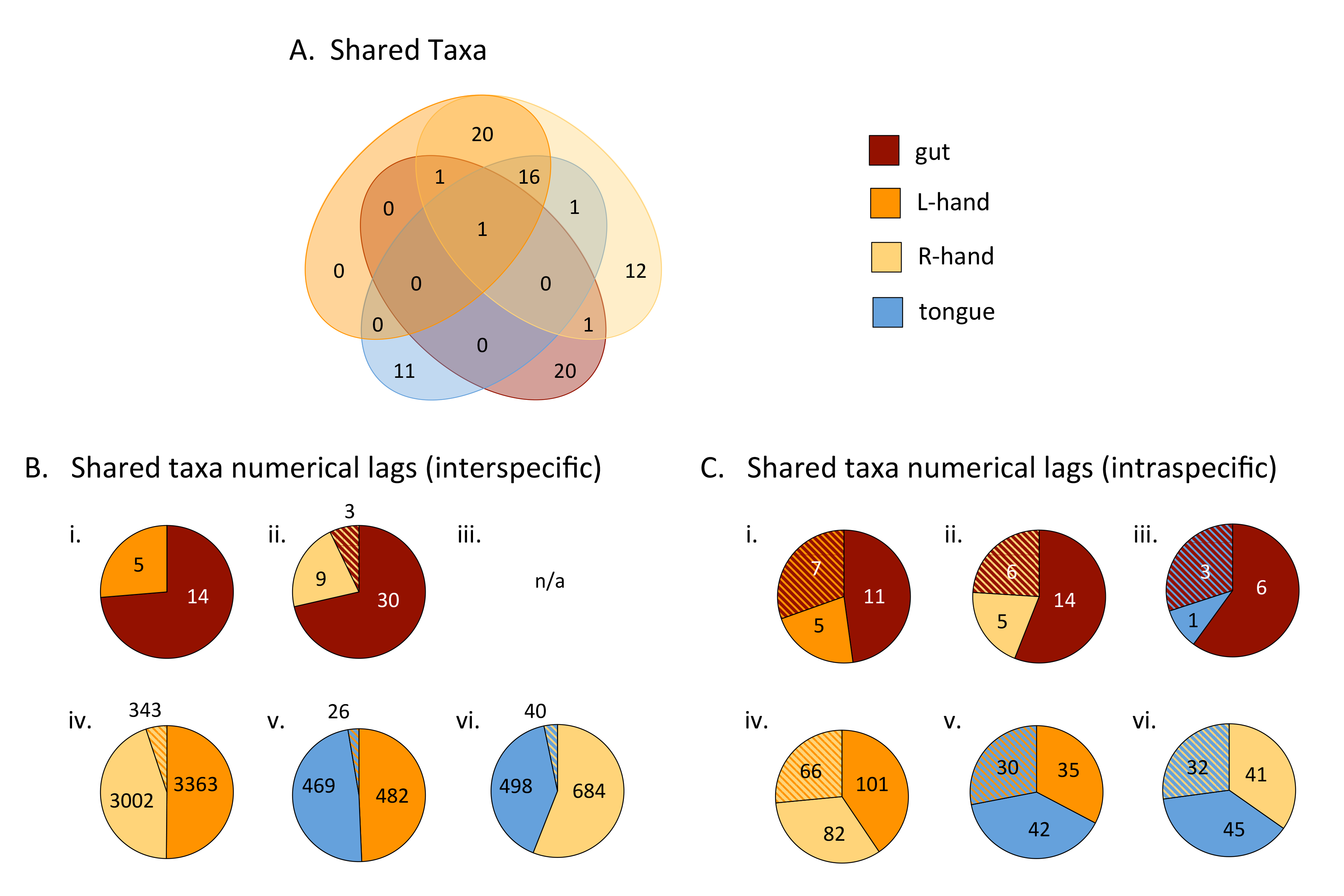

Supplement: S2 Fig — (A) Venn diagram showing the number of shared genera amongst the gut, left-hand, right-hand and tongue; (B,C) Pie charts for pairwise combinations of body sites illustrating the number of time-lags with significant, taxon-specific coefficients that are unique to one or other body site (solid) or else shared between body sites (striped). For each chart, we include only those taxa found on both body sites being compared (see A) and treat positive and negative coefficients separately (i.e., to be classified as a shared time-lag, the sign must be the same). Individual panels are as follows: (B) interspecific interactions considering all time-lags from 1 to 20 days, (C) intraspecific interactions considering all time-lags from 1 to 20 days. (TIF) [file pcbi.1007037.s016.tif]

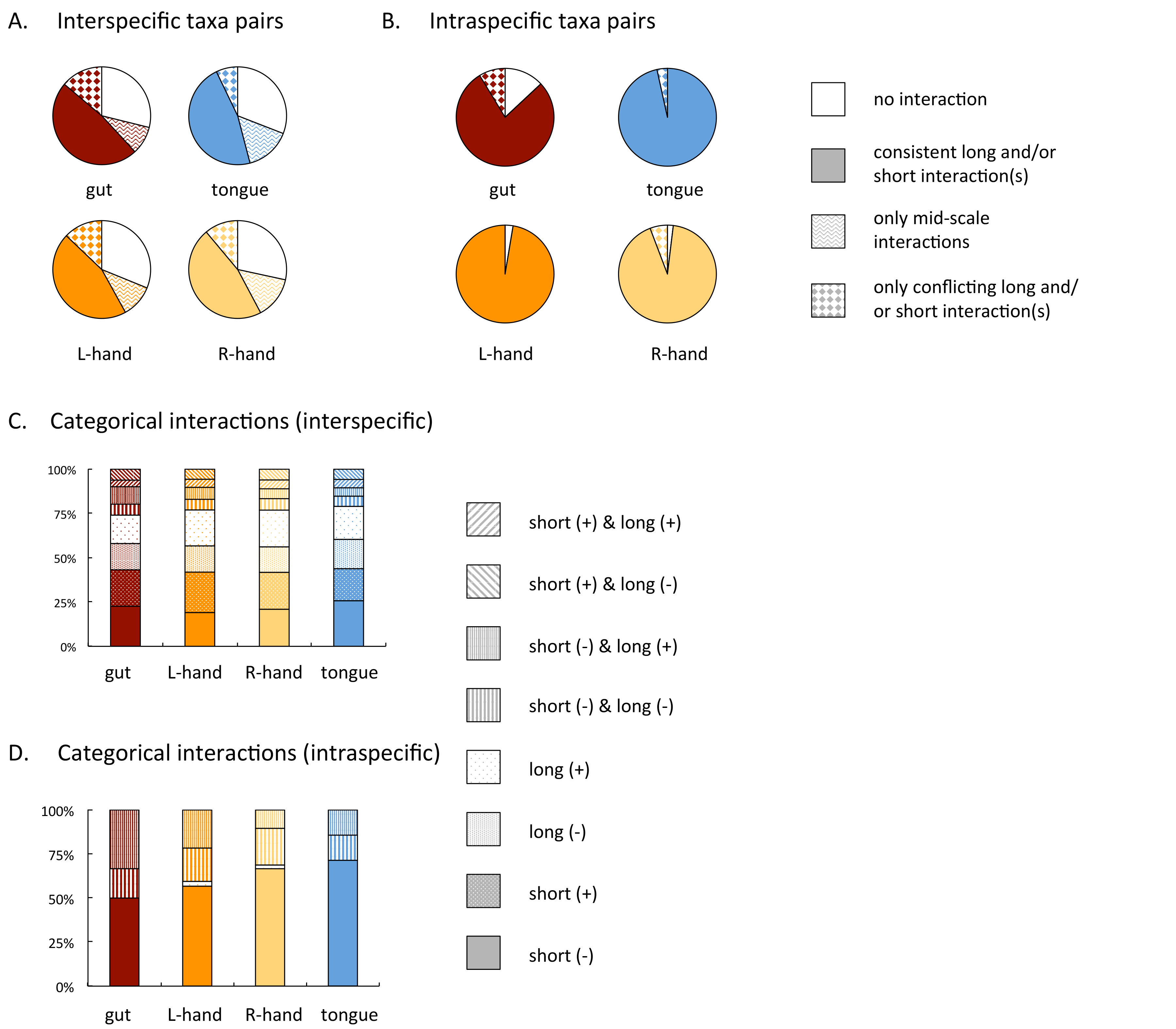

Supplement: S3 Fig — (A,B) Fraction of taxon pairs at the four body sites that exhibit no interaction (white), consistent long and/or short interactions (solid), only interactions between 11–14 days (wavy lines) and only short and/or long interactions with conflicting signs for (A) interspecific interactions and (B) intraspecific interactions. (C,D) Fraction of consistent short and/or long interacting taxon pairs with various combinations of negative and positive short and long interactions for (C) interspecific interactions and (D) intraspecific interactions. (TIF) [file pcbi.1007037.s017.tif]

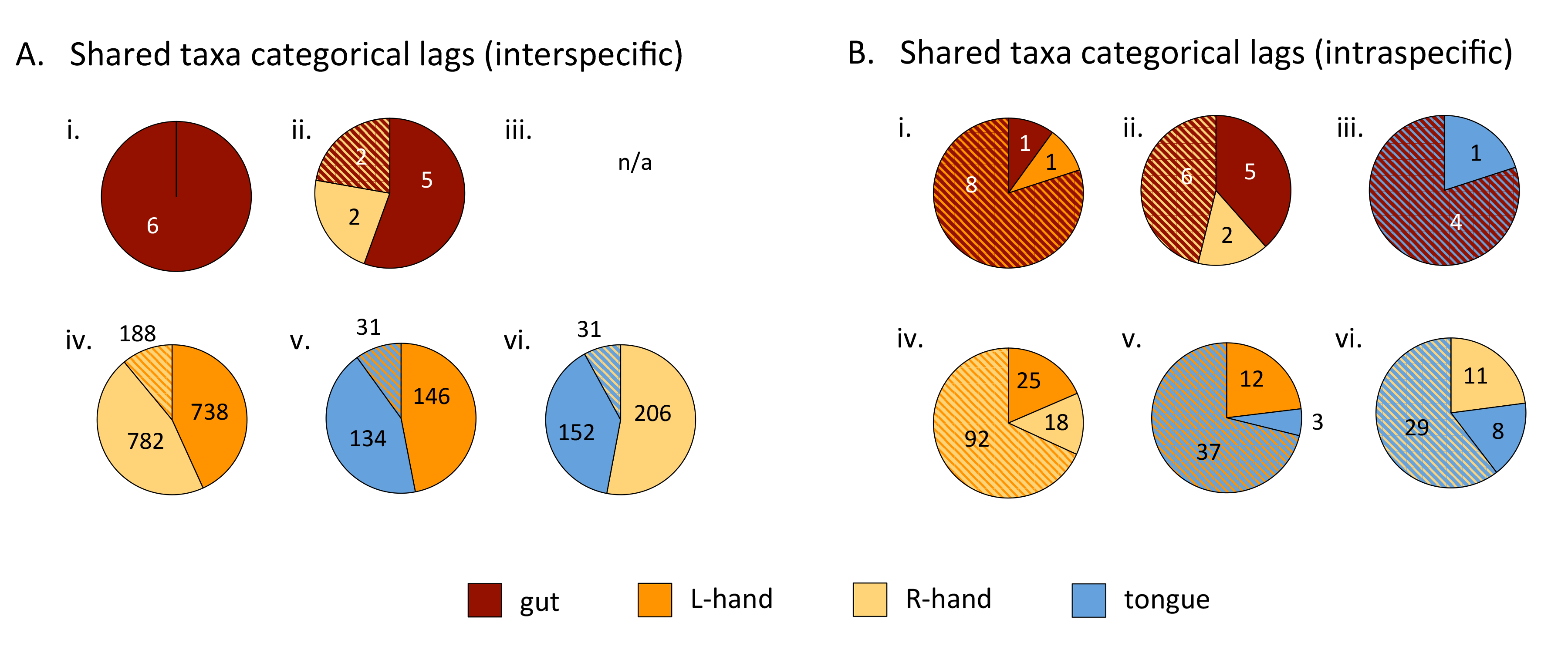

Supplement: S4 Fig — For each chart, we include only those taxa found on both body sites being compared (see S2 Fig, panel A) and treat positive and negative coefficients separately (i.e., to be classified as a shared time-lag, the sign must be the same). Individual panels are as follows: (A) interspecific interactions considering only ‘short’ and ‘long’ timescales and (B) intraspecific interactions considering only ‘short’ and ‘long’ timescales. (TIF) [file pcbi.1007037.s018.tif]
